# Supplementary material for: Multiple immunity-related genes control susceptibility of Arabidopsis thaliana to the parasitic weed Phelipanche aegyptiaca
Source: PeerJ. 2020 Jun 8;8:e9268. doi: 10.7717/peerj.9268 (PMC7289146; doi:10.7717/peerj.9268)
Supplement: Supplemental Information 1 [file peerj-08-9268-s001.docx]

**Supplementary Table 1.** Type III test for fixed effects for the factors plant, position, and bag on attachment odds ratios.

|  |  |  | Initial attachment | | Early tubercle | | Late tubercle | |
| --- | --- | --- | --- | --- | --- | --- | --- | --- |
| Factor | Num DF | Den DF | F Value | PR > F | F Value | PR > F | F Value | PR > F |
| **Plant** | 43 | 836 | 1.76 | 0.0022 | 2.85 | <.0001 | 3.79 | <.0001 |
| **Bag** | 7 | 836 | 1 | 0.4304 | 1.48 | 0.1714 | 1.48 | 0.1718 |
| **Position** | 4 | 836 | 0.63 | 0.6443 | 2.74 | 0.0278 | 3.12 | 0.0146 |
