# Supplementary material for: Multiple immunity-related genes control susceptibility of Arabidopsis thaliana to the parasitic weed Phelipanche aegyptiaca
Source: PeerJ. 2020 Jun 8;8:e9268. doi: 10.7717/peerj.9268 (PMC7289146; doi:10.7717/peerj.9268)
Supplement: Supplemental Information 2 [file peerj-08-9268-s002.docx]

**Supplementary Table 2.** qPCR primers used in this study.

| **Gene** | **Pathway** | **Fwd Primer** | **Reverse primer** | **Citation** |
| --- | --- | --- | --- | --- |
| *VSP2* | JA | CAAACTAAACAATAAACCATACCATAA | GCCAAGAGCAAGAGAAGTGA | [1] |
| *IAA13* | Auxin | CACGAAATCAAGAACCAAACGA | CACCGTAACGTCGAAAAGAGATC | [2] |
| *IAA2* | Auxin | GGTTGGCCACCAGTGAGATC | AGCTCCGTCCATACTCACTTTCA | [2] |
| *MYB51* | Glucosinolates | ACCAACCTCGAATCTTCTCTG | TTTCAACACAAGACTCCTCCA | [1] |
| *EXPPT1* | GA | GGATTTCATTCGTCAAACCT | CAACCAATATCAAAGCGGAG | [1] |
| *ARR10* | CK | GCTTCTGATGCTGGTTCCTT | CAATCACCTTCCGAGAAATCA | [3] |
| *ACS6* | Ethylene | TTATCTCAGCGTGCCTTGCA | CCGATGAAGAGTTTGTAGACGAGTT | [2] |
| *BOI* | Neg. cell death reg. | TCTTCGAACAAACCTAGACC | CACAAACCGTACACAAACAC | [1] |
| *SAND* | Reference | CTGTCTTCTCATCTCTTGTC | TCTTGCAATATGGTTCCTG | [4] |
| *UBQ10* | Reference | GAAGTTCAATGTTTCGTTTCATGT | GGATTATACAAGGCCCCAAAA | [3] |
| *UFP* | Reference | CCAGCAGACATGGAGGTTTTGGGG | TGTTGTCTGTCATTTCTTGGCCAGT | [5] |
| *PAD4* | defense/SA | ACCGAGGAACATCAGAGGTAC | AAATTCGCAATGTCGAGTGGC | [6] |
| *SID2* | SA | TCCGTGACCTTGATCCTTTC | ACAGCGATCTTGCCATTAGG | [1] |
| *PR1* | SA | CTCATACACTCTGGTGGG | TTGGCACATCCGAGTC | [7] |
| *WRKY53* | PTI | GCAACGAAACAAGTCCAGAG | GTCTTTACCATCATCAAGCCC | [1] |
