# Supplementary material for: Multiple immunity-related genes control susceptibility of Arabidopsis thaliana to the parasitic weed Phelipanche aegyptiaca
Source: PeerJ. 2020 Jun 8;8:e9268. doi: 10.7717/peerj.9268 (PMC7289146; doi:10.7717/peerj.9268)
Supplement: Supplemental Information 3 [file peerj-08-9268-s003.docx]

**Supplementary Table 3.** Detailed results from all attachment rate experiments.

| **Plant** | **Bag** | **Position** | **Unattached** | **S3** | **S4.1** | **S4.2** | **S3 rate** | **S4 Rate** | **S4.2 Rate** | **Experiment block** |
| --- | --- | --- | --- | --- | --- | --- | --- | --- | --- | --- |
| *35s::ERF2* | 1 | 4 | 30 | 0 | 0 | 3 | 0.090909 | 0.090909091 | 0.090909 | 25 |
| *35s::ERF2* | 4 | 3 | 19 | 0 | 2 | 1 | 0.136364 | 0.136363636 | 0.045455 | 25 |
| *35s::ERF2* | 5 | 4 | 35 | 0 | 1 | 2 | 0.078947 | 0.078947368 | 0.052632 | 25 |
| *35s::ERF2* | 1 | 5 | 26 | 0 | 0 | 0 | 0 | 0 | 0 | 34 |
| *35s::ERF2* | 3 | 4 | 11 | 1 | 3 | 6 | 0.47619 | 0.428571429 | 0.285714 | 34 |
| *35s::ERF2* | 6 | 5 | 28 | 0 | 0 | 0 | 0 | 0 | 0 | 34 |
| *35s::ERF2* | 7 | 2 | 16 | 0 | 3 | 8 | 0.407407 | 0.407407407 | 0.296296 | 34 |
| *35s::ERF2* | 1 | 4 | 30 | 0 | 3 | 1 | 0.117647 | 0.117647059 | 0.029412 | 22 |
| *35s::ERF2* | 2 | 5 | 34 | 0 | 4 | 7 | 0.244444 | 0.244444444 | 0.155556 | 22 |
| *35s::ERF2* | 3 | 4 | 49 | 0 | 6 | 7 | 0.209677 | 0.209677419 | 0.112903 | 22 |
| *35s::ERF2* | 4 | 3 | 35 | 0 | 1 | 3 | 0.102564 | 0.102564103 | 0.076923 | 22 |
| *35s::ERF2* | 5 | 4 | 27 | 0 | 0 | 2 | 0.068966 | 0.068965517 | 0.068966 | 22 |
| *35s::ERF2* | 1 | 1 | 19 | 0 | 0 | 4 | 0.173913 | 0.173913043 | 0.173913 | 2 |
| *35s::ERF2* | 3 | 1 | 25 | 0 | 1 | 2 | 0.107143 | 0.107142857 | 0.071429 | 2 |
| *35s::ERF2* | 5 | 4 | 16 | 0 | 4 | 3 | 0.304348 | 0.304347826 | 0.130435 | 2 |
| *35s::PMR4* | 1 | 5 | 23 | 0 | 2 | 2 | 0.148148 | 0.148148148 | 0.074074 | 14 |
| *35s::PMR4* | 3 | 5 | 32 | 1 | 0 | 4 | 0.135135 | 0.108108108 | 0.108108 | 14 |
| *35s::PMR4* | 4 | 2 | 15 | 0 | 0 | 4 | 0.210526 | 0.210526316 | 0.210526 | 14 |
| *35s::PMR4* | 6 | 2 | 36 | 0 | 0 | 2 | 0.052632 | 0.052631579 | 0.052632 | 14 |
| *35s::PMR4* | 7 | 1 | 35 | 1 | 1 | 0 | 0.054054 | 0.027027027 | 0 | 14 |
| *35s::PMR4* | 7 | 4 | 24 | 0 | 3 | 4 | 0.225806 | 0.225806452 | 0.129032 | 14 |
| *35s::PMR4* | 8 | 4 | 24 | 2 | 2 | 10 | 0.368421 | 0.315789474 | 0.263158 | 14 |
| *35s::PMR4* | 1 | 1 | 20 | 0 | 1 | 1 | 0.090909 | 0.090909091 | 0.045455 | 6 |
| *35s::PMR4* | 2 | 1 | 16 | 0 | 2 | 0 | 0.111111 | 0.111111111 | 0 | 6 |
| *35s::PMR4* | 4 | 3 | 22 | 1 | 0 | 0 | 0.043478 | 0 | 0 | 6 |
| *35s::PMR4* | 5 | 1 | 18 | 1 | 3 | 0 | 0.181818 | 0.136363636 | 0 | 6 |
| *35s::PMR4* | 6 | 4 | 25 | 2 | 3 | 1 | 0.193548 | 0.129032258 | 0.032258 | 6 |
| *35s::PMR4* | 7 | 4 | 27 | 0 | 0 | 1 | 0.035714 | 0.035714286 | 0.035714 | 6 |
| *35s::PMR4* | 8 | 2 | 22 | 0 | 0 | 0 | 0 | 0 | 0 | 6 |
| *35s::PMR4* | 1 | 4 | 9 | 0 | 1 | 0 | 0.1 | 0.1 | 0 | 19 |
| *35s::PMR4* | 2 | 3 | 28 | 2 | 2 | 0 | 0.125 | 0.0625 | 0 | 19 |
| *35s::PMR4* | 3 | 5 | 16 | 0 | 1 | 4 | 0.238095 | 0.238095238 | 0.190476 | 19 |
| *35s::PMR4* | 4 | 2 | 16 | 1 | 0 | 0 | 0.058824 | 0 | 0 | 19 |
| *35s::PMR4* | 5 | 5 | 6 | 0 | 3 | 0 | 0.333333 | 0.333333333 | 0 | 19 |
| *35s::PMR4* | 6 | 1 | 14 | 0 | 0 | 4 | 0.222222 | 0.222222222 | 0.222222 | 19 |
| *35s::PMR4* | 7 | 2 | 9 | 0 | 0 | 2 | 0.181818 | 0.181818182 | 0.181818 | 19 |
| *35s::PMR4* | 8 | 4 | 13 | 0 | 1 | 1 | 0.133333 | 0.133333333 | 0.066667 | 19 |
| *aba1* |  |  | 14 | 0 | 1 | 0 | 0.066667 | 0.066666667 | 0 | 4 |
| *aba1* |  |  | 31 | 2 | 7 | 4 | 0.295455 | 0.25 | 0.090909 | 4 |
| *aba1* |  |  | 30 | 0 | 6 | 1 | 0.189189 | 0.189189189 | 0.027027 | 4 |
| *aba1* |  |  | 17 | 2 | 4 | 1 | 0.291667 | 0.208333333 | 0.041667 | 4 |
| *aba1* |  |  | 20 | 1 | 4 | 1 | 0.230769 | 0.192307692 | 0.038462 | 4 |
| *aba1* |  |  | 27 | 1 | 1 | 3 | 0.15625 | 0.125 | 0.09375 | 4 |
| *aba1* | 2 | 3 | 27 | 1 | 1 | 2 | 0.129032 | 0.096774194 | 0.064516 | 27 |
| *aba1* | 3 | 1 | 23 | 1 | 1 | 1 | 0.115385 | 0.076923077 | 0.038462 | 27 |
| *aba1* | 5 | 2 | 17 | 1 | 3 | 7 | 0.392857 | 0.357142857 | 0.25 | 27 |
| *aba1* | 6 | 5 | 28 | 0 | 1 | 0 | 0.034483 | 0.034482759 | 0 | 27 |
| *aba1* | 7 | 4 | 41 | 0 | 3 | 5 | 0.163265 | 0.163265306 | 0.102041 | 27 |
| *aba1* | 8 | 4 | 33 | 0 | 1 | 1 | 0.057143 | 0.057142857 | 0.028571 | 27 |
| *aba1* | 1 | 2 | 14 | 0 | 2 | 3 | 0.263158 | 0.263157895 | 0.157895 | 6 |
| *aba1* | 2 | 4 | 22 | 1 | 1 | 0 | 0.083333 | 0.041666667 | 0 | 6 |
| *aba1* | 4 | 1 | 19 | 0 | 1 | 2 | 0.136364 | 0.136363636 | 0.090909 | 6 |
| *aba1* | 5 | 2 | 20 | 2 | 0 | 0 | 0.090909 | 0 | 0 | 6 |
| *aba1* | 6 | 3 | 26 | 0 | 0 | 3 | 0.103448 | 0.103448276 | 0.103448 | 6 |
| *aba1* | 7 | 2 | 24 | 0 | 2 | 0 | 0.076923 | 0.076923077 | 0 | 6 |
| *aba1* | 8 | 4 | 25 | 0 | 0 | 0 | 0 | 0 | 0 | 6 |
| *aba1* | 1 | 3 | 26 | 2 | 4 | 2 | 0.235294 | 0.176470588 | 0.058824 | 23 |
| *aba1* | 2 | 4 | 31 | 0 | 2 | 4 | 0.162162 | 0.162162162 | 0.108108 | 23 |
| *aba1* | 3 | 4 | 19 | 1 | 1 | 2 | 0.173913 | 0.130434783 | 0.086957 | 23 |
| *aba1* | 5 | 1 | 23 | 0 | 0 | 1 | 0.041667 | 0.041666667 | 0.041667 | 23 |
| *aba1* | 6 | 4 | 18 | 0 | 4 | 1 | 0.217391 | 0.217391304 | 0.043478 | 23 |
| *aba1* | 7 | 3 | 27 | 0 | 1 | 0 | 0.035714 | 0.035714286 | 0 | 23 |
| *aux1-7* | 1 | 2 | 17 | 0 | 2 | 2 | 0.190476 | 0.19047619 | 0.095238 | 2 |
| *aux1-7* | 2 | 5 | 12 | 0 | 3 | 1 | 0.25 | 0.25 | 0.0625 | 2 |
| *aux1-7* | 3 | 4 | 20 | 0 | 0 | 3 | 0.130435 | 0.130434783 | 0.130435 | 2 |
| *aux1-7* | 5 | 5 | 18 | 1 | 0 | 1 | 0.1 | 0.05 | 0.05 | 2 |
| *aux1-7* | 6 | 3 | 22 | 2 | 4 | 2 | 0.266667 | 0.2 | 0.066667 | 2 |
| *aux1-7* | 1 | 4 | 16 | 0 | 1 | 3 | 0.2 | 0.2 | 0.15 | 6 |
| *aux1-7* | 2 | 2 | 21 | 1 | 1 | 0 | 0.086957 | 0.043478261 | 0 | 6 |
| *aux1-7* | 3 | 3 | 16 | 1 | 1 | 2 | 0.2 | 0.15 | 0.1 | 6 |
| *aux1-7* | 4 | 2 | 11 | 0 | 0 | 2 | 0.153846 | 0.153846154 | 0.153846 | 6 |
| *aux1-7* | 5 | 4 | 23 | 0 | 1 | 0 | 0.041667 | 0.041666667 | 0 | 6 |
| *aux1-7* | 7 | 1 | 21 | 0 | 1 | 2 | 0.125 | 0.125 | 0.083333 | 6 |
| *aux1-7* | 8 | 1 | 16 | 0 | 3 | 1 | 0.2 | 0.2 | 0.05 | 6 |
| *aux1-7* | 1 | 5 | 12 | 0 | 2 | 1 | 0.2 | 0.2 | 0.066667 | 8 |
| *aux1-7* | 2 | 5 | 14 | 1 | 3 | 1 | 0.263158 | 0.210526316 | 0.052632 | 8 |
| *aux1-7* | 3 | 3 | 15 | 0 | 2 | 1 | 0.166667 | 0.166666667 | 0.055556 | 8 |
| *aux1-7* | 4 | 4 | 14 | 0 | 3 | 2 | 0.263158 | 0.263157895 | 0.105263 | 8 |
| *aux1-7* | 5 | 1 | 8 | 1 | 2 | 6 | 0.529412 | 0.470588235 | 0.352941 | 8 |
| *aux1-7* | 6 | 2 | 11 | 0 | 0 | 2 | 0.153846 | 0.153846154 | 0.153846 | 8 |
| *aux1-7* | 7 | 4 | 14 | 1 | 0 | 1 | 0.125 | 0.0625 | 0.0625 | 8 |
| *aux1-7/ein2-1* | 2 | 4 | 27 | 0 | 9 | 3 | 0.307692 | 0.307692308 | 0.076923 | 3 |
| *aux1-7/ein2-1* | 4 | 2 | 22 | 0 | 5 | 1 | 0.214286 | 0.214285714 | 0.035714 | 3 |
| *aux1-7/ein2-1* | 4 | 5 | 22 | 3 | 1 | 1 | 0.185185 | 0.074074074 | 0.037037 | 3 |
| *aux1-7/ein2-1* | 5 | 1 | 22 | 0 | 1 | 1 | 0.083333 | 0.083333333 | 0.041667 | 3 |
| *aux1-7/ein2-1* | 6 | 4 | 18 | 2 | 4 | 2 | 0.307692 | 0.230769231 | 0.076923 | 3 |
| *aux1-7/ein2-1* | 1 | 5 | 20 | 1 | 1 | 7 | 0.310345 | 0.275862069 | 0.241379 | 18 |
| *aux1-7/ein2-1* | 2 | 1 | 26 | 0 | 1 | 2 | 0.103448 | 0.103448276 | 0.068966 | 18 |
| *aux1-7/ein2-1* | 3 | 1 | 44 | 0 | 2 | 4 | 0.12 | 0.12 | 0.08 | 18 |
| *aux1-7/ein2-1* | 4 | 4 | 14 | 3 | 2 | 4 | 0.391304 | 0.260869565 | 0.173913 | 18 |
| *aux1-7/ein2-1* | 8 | 4 | 17 | 1 | 0 | 7 | 0.32 | 0.28 | 0.28 | 18 |
| *aux1-7/ein2-1* | 3 | 3 | 36 | 0 | 2 | 5 | 0.162791 | 0.162790698 | 0.116279 | 20 |
| *aux1-7/ein2-1* | 4 | 1 | 24 | 0 | 6 | 2 | 0.25 | 0.25 | 0.0625 | 20 |
| *aux1-7/ein2-1* | 5 | 1 | 33 | 1 | 5 | 4 | 0.232558 | 0.209302326 | 0.093023 | 20 |
| *aux1-7/ein2-1* | 6 | 5 | 23 | 1 | 8 | 7 | 0.410256 | 0.384615385 | 0.179487 | 20 |
| *aux1-7/ein2-1* | 7 | 2 | 8 | 1 | 0 | 0 | 0.111111 | 0 | 0 | 20 |
| *aux1-7/ein2-1* | 8 | 5 | 34 | 1 | 2 | 1 | 0.105263 | 0.078947368 | 0.026316 | 20 |
| *axr1-3* | 1 | 2 | 29 | 0 | 3 | 3 | 0.171429 | 0.171428571 | 0.085714 | 11 |
| *axr1-3* | 2 | 3 | 32 | 0 | 0 | 0 | 0 | 0 | 0 | 11 |
| *axr1-3* | 3 | 3 | 25 | 0 | 0 | 0 | 0 | 0 | 0 | 11 |
| *axr1-3* | 4 | 5 | 27 | 0 | 0 | 0 | 0 | 0 | 0 | 11 |
| *axr1-3* | 5 | 4 | 23 | 0 | 0 | 0 | 0 | 0 | 0 | 11 |
| *axr1-3* | 6 | 2 | 28 | 0 | 0 | 4 | 0.125 | 0.125 | 0.125 | 11 |
| *axr1-3* | 1 | 4 | 16 | 0 | 2 | 4 | 0.272727 | 0.272727273 | 0.181818 | 20 |
| *axr1-3* | 2 | 3 | 19 | 1 | 1 | 4 | 0.24 | 0.2 | 0.16 | 20 |
| *axr1-3* | 3 | 2 | 29 | 1 | 3 | 3 | 0.194444 | 0.166666667 | 0.083333 | 20 |
| *axr1-3* | 4 | 5 | 20 | 0 | 0 | 3 | 0.130435 | 0.130434783 | 0.130435 | 20 |
| *axr1-3* | 5 | 2 | 19 | 1 | 1 | 4 | 0.24 | 0.2 | 0.16 | 20 |
| *axr1-3* | 6 | 2 | 22 | 2 | 5 | 2 | 0.290323 | 0.225806452 | 0.064516 | 20 |
| *axr1-3* | 7 | 5 | 20 | 1 | 1 | 2 | 0.166667 | 0.125 | 0.083333 | 20 |
| *axr1-3* | 8 | 2 | 19 | 0 | 0 | 1 | 0.05 | 0.05 | 0.05 | 20 |
| *bak1-4* | 1 | 5 | 17 | 0 | 1 | 1 | 0.105263 | 0.105263158 | 0.052632 | 9 |
| *bak1-4* | 3 | 2 | 32 | 0 | 10 | 0 | 0.238095 | 0.238095238 | 0 | 9 |
| *bak1-4* | 4 | 4 | 19 | 4 | 6 | 1 | 0.366667 | 0.233333333 | 0.033333 | 9 |
| *bak1-4* | 5 | 4 | 18 | 3 | 7 | 2 | 0.4 | 0.3 | 0.066667 | 9 |
| *bak1-4* | 5 | 5 | 13 | 0 | 5 | 0 | 0.277778 | 0.277777778 | 0 | 9 |
| *bak1-4* | 6 | 3 | 15 | 4 | 2 | 5 | 0.423077 | 0.269230769 | 0.192308 | 9 |
| *bak1-4* | 1 | 3 | 17 | 0 | 1 | 1 | 0.105263 | 0.105263158 | 0.052632 | 19 |
| *bak1-4* | 2 | 5 | 20 | 2 | 3 | 1 | 0.230769 | 0.153846154 | 0.038462 | 19 |
| *bak1-4* | 4 | 4 | 17 | 0 | 0 | 1 | 0.055556 | 0.055555556 | 0.055556 | 19 |
| *bak1-4* | 6 | 5 | 13 | 1 | 3 | 6 | 0.434783 | 0.391304348 | 0.26087 | 19 |
| *bak1-4* | 7 | 3 | 15 | 0 | 3 | 2 | 0.25 | 0.25 | 0.1 | 19 |
| *bak1-4* | 8 | 5 | 7 | 0 | 0 | 1 | 0.125 | 0.125 | 0.125 | 19 |
| *bak1-4* | 1 | 1 | 26 | 0 | 2 | 0 | 0.071429 | 0.071428571 | 0 | 31 |
| *bak1-4* | 4 | 3 | 23 | 0 | 0 | 1 | 0.041667 | 0.041666667 | 0.041667 | 31 |
| *bak1-4* | 6 | 4 | 31 | 1 | 2 | 3 | 0.162162 | 0.135135135 | 0.081081 | 31 |
| *bkk1* | 1 | 5 | 20 | 0 | 2 | 3 | 0.2 | 0.2 | 0.12 | 10 |
| *bkk1* | 3 | 1 | 18 | 0 | 0 | 1 | 0.052632 | 0.052631579 | 0.052632 | 10 |
| *bkk1* | 4 | 1 | 26 | 0 | 2 | 0 | 0.071429 | 0.071428571 | 0 | 10 |
| *bkk1* | 5 | 2 | 25 | 0 | 6 | 1 | 0.21875 | 0.21875 | 0.03125 | 10 |
| *bkk1* | 6 | 3 | 22 | 1 | 0 | 2 | 0.12 | 0.08 | 0.08 | 10 |
| *bkk1* | 2 | 3 | 5 | 0 | 0 | 0 | 0 | 0 | 0 | 12 |
| *bkk1* | 6 | 3 | 43 | 0 | 2 | 0 | 0.044444 | 0.044444444 | 0 | 12 |
| *bkk1* | 1 | 2 | 26 | 2 | 0 | 2 | 0.133333 | 0.066666667 | 0.066667 | 18 |
| *bkk1* | 3 | 2 | 19 | 0 | 0 | 6 | 0.24 | 0.24 | 0.24 | 18 |
| *bkk1* | 4 | 1 | 30 | 2 | 0 | 4 | 0.166667 | 0.111111111 | 0.111111 | 18 |
| *bkk1* | 5 | 2 | 24 | 1 | 0 | 6 | 0.225806 | 0.193548387 | 0.193548 | 18 |
| *bkk1* | 6 | 4 | 25 | 2 | 5 | 4 | 0.305556 | 0.25 | 0.111111 | 18 |
| *bkk1* | 7 | 5 | 40 | 0 | 4 | 5 | 0.183673 | 0.183673469 | 0.102041 | 18 |
| *bkk1* | 8 | 3 | 22 | 0 | 2 | 7 | 0.290323 | 0.290322581 | 0.225806 | 18 |
| *bkk1* | 3 | 4 | 21 | 0 | 0 | 2 | 0.086957 | 0.086956522 | 0.086957 | 41 |
| *bkk1* | 4 | 4 | 24 | 0 | 1 | 2 | 0.111111 | 0.111111111 | 0.074074 | 41 |
| *bkk1* | 6 | 2 | 14 | 0 | 1 | 1 | 0.125 | 0.125 | 0.0625 | 41 |
| *bkk1* | 8 | 2 | 19 | 1 | 2 | 0 | 0.136364 | 0.090909091 | 0 | 41 |
| Col | 6 | 1 | 31 | 0 | 0 | 1 | 0.03125 | 0.03125 | 0.03125 | 11 |
| Col | 1 | 4 | 21 | 1 | 1 | 6 | 0.275862 | 0.24137931 | 0.206897 | 11 |
| Col | 2 | 4 | 33 | 2 | 4 | 3 | 0.214286 | 0.166666667 | 0.071429 | 11 |
| Col | 3 | 4 | 17 | 0 | 1 | 1 | 0.105263 | 0.105263158 | 0.052632 | 11 |
| Col | 4 | 2 | 27 | 1 | 2 | 0 | 0.1 | 0.066666667 | 0 | 11 |
| Col | 5 | 2 | 18 | 1 | 0 | 0 | 0.052632 | 0 | 0 | 11 |
| Col | 1 | 4 | 22 | 1 | 0 | 1 | 0.083333 | 0.041666667 | 0.041667 | 30 |
| Col | 2 | 5 | 22 | 1 | 1 | 0 | 0.083333 | 0.041666667 | 0 | 30 |
| Col | 4 | 2 | 23 | 0 | 3 | 3 | 0.206897 | 0.206896552 | 0.103448 | 30 |
| Col | 7 | 1 | 23 | 2 | 2 | 1 | 0.178571 | 0.107142857 | 0.035714 | 30 |
| Col | 8 | 3 | 13 | 0 | 2 | 2 | 0.235294 | 0.235294118 | 0.117647 | 30 |
| Col | 1 | 5 | 26 | 0 | 5 | 4 | 0.257143 | 0.257142857 | 0.114286 | 2 |
| Col | 3 | 5 | 17 | 0 | 5 | 5 | 0.37037 | 0.37037037 | 0.185185 | 2 |
| Col | 4 | 4 | 20 | 0 | 2 | 2 | 0.166667 | 0.166666667 | 0.083333 | 2 |
| Col | 5 | 5 | 19 | 0 | 1 | 1 | 0.095238 | 0.095238095 | 0.047619 | 2 |
| Col | 6 | 2 | 20 | 1 | 2 | 0 | 0.130435 | 0.086956522 | 0 | 3 |
| Col | 1 | 3 | 10 | 0 | 1 | 2 | 0.230769 | 0.230769231 | 0.153846 | 3 |
| Col | 2 | 3 | 20 | 0 | 8 | 2 | 0.333333 | 0.333333333 | 0.066667 | 3 |
| Col | 2 | 5 | 22 | 1 | 6 | 2 | 0.290323 | 0.258064516 | 0.064516 | 3 |
| Col | 3 | 1 | 17 | 0 | 1 | 3 | 0.190476 | 0.19047619 | 0.142857 | 3 |
| Col | 4 | 3 | 25 | 0 | 5 | 1 | 0.193548 | 0.193548387 | 0.032258 | 3 |
| Col | 5 | 4 | 20 | 0 | 3 | 3 | 0.230769 | 0.230769231 | 0.115385 | 3 |
| Col |  |  | 19 | 2 | 0 | 0 | 0.095238 | 0 | 0 | 4 |
| Col |  |  | 17 | 0 | 2 | 1 | 0.15 | 0.15 | 0.05 | 4 |
| Col |  |  | 26 | 1 | 5 | 1 | 0.212121 | 0.181818182 | 0.030303 | 4 |
| Col |  |  | 27 | 1 | 4 | 1 | 0.181818 | 0.151515152 | 0.030303 | 4 |
| Col |  |  | 21 | 0 | 5 | 2 | 0.25 | 0.25 | 0.071429 | 4 |
| Col |  |  | 28 | 2 | 5 | 2 | 0.243243 | 0.189189189 | 0.054054 | 4 |
| Col | 2 | 3 | 26 | 0 | 4 | 0 | 0.133333 | 0.133333333 | 0 | 6 |
| Col | 4 | 4 | 20 | 1 | 1 | 0 | 0.090909 | 0.045454545 | 0 | 6 |
| Col | 5 | 3 | 24 | 0 | 5 | 1 | 0.2 | 0.2 | 0.033333 | 6 |
| Col | 6 | 1 | 23 | 0 | 1 | 2 | 0.115385 | 0.115384615 | 0.076923 | 6 |
| Col | 7 | 3 | 15 | 0 | 1 | 2 | 0.166667 | 0.166666667 | 0.111111 | 6 |
| Col | 8 | 3 | 23 | 0 | 1 | 0 | 0.041667 | 0.041666667 | 0 | 6 |
| Col | 1 | 2 | 28 | 0 | 2 | 1 | 0.096774 | 0.096774194 | 0.032258 | 7 |
| Col | 3 | 3 | 18 | 0 | 3 | 1 | 0.181818 | 0.181818182 | 0.045455 | 7 |
| Col | 4 | 2 | 15 | 1 | 4 | 0 | 0.25 | 0.2 | 0 | 7 |
| Col | 5 | 3 | 31 | 0 | 0 | 1 | 0.03125 | 0.03125 | 0.03125 | 7 |
| Col | 6 | 5 | 22 | 2 | 6 | 1 | 0.290323 | 0.225806452 | 0.032258 | 7 |
| Col | 1 | 1 | 19 | 0 | 2 | 0 | 0.095238 | 0.095238095 | 0 | 8 |
| Col | 3 | 2 | 14 | 0 | 0 | 3 | 0.176471 | 0.176470588 | 0.176471 | 8 |
| Col | 4 | 2 | 9 | 0 | 0 | 1 | 0.1 | 0.1 | 0.1 | 8 |
| Col | 5 | 4 | 9 | 0 | 2 | 2 | 0.307692 | 0.307692308 | 0.153846 | 8 |
| Col | 6 | 4 | 14 | 0 | 1 | 6 | 0.333333 | 0.333333333 | 0.285714 | 8 |
| Col | 7 | 5 | 13 | 1 | 2 | 3 | 0.315789 | 0.263157895 | 0.157895 | 8 |
| Col | 6 | 4 | 44 | 0 | 3 | 1 | 0.083333 | 0.083333333 | 0.020833 | 9 |
| Col | 1 | 4 | 17 | 3 | 4 | 2 | 0.346154 | 0.230769231 | 0.076923 | 9 |
| Col | 2 | 3 | 7 | 3 | 1 | 2 | 0.461538 | 0.230769231 | 0.153846 | 9 |
| Col | 2 | 5 | 24 | 0 | 3 | 2 | 0.172414 | 0.172413793 | 0.068966 | 9 |
| Col | 3 | 1 | 23 | 0 | 5 | 1 | 0.206897 | 0.206896552 | 0.034483 | 9 |
| Col | 3 | 4 | 24 | 0 | 3 | 2 | 0.172414 | 0.172413793 | 0.068966 | 9 |
| Col | 4 | 1 | 6 | 0 | 2 | 0 | 0.25 | 0.25 | 0 | 9 |
| Col | 4 | 5 | 28 | 0 | 3 | 0 | 0.096774 | 0.096774194 | 0 | 9 |
| Col | 5 | 3 | 29 | 2 | 9 | 0 | 0.275 | 0.225 | 0 | 9 |
| Col | 6 | 1 | 33 | 0 | 3 | 1 | 0.108108 | 0.108108108 | 0.027027 | 9 |
| Col | 1 | 1 | 13 | 1 | 0 | 0 | 0.071429 | 0 | 0 | 10 |
| Col | 3 | 3 | 22 | 1 | 3 | 0 | 0.153846 | 0.115384615 | 0 | 10 |
| Col | 4 | 3 | 15 | 0 | 2 | 1 | 0.166667 | 0.166666667 | 0.055556 | 10 |
| Col | 5 | 5 | 36 | 0 | 1 | 2 | 0.076923 | 0.076923077 | 0.051282 | 10 |
| Col | 6 | 4 | 20 | 1 | 2 | 0 | 0.130435 | 0.086956522 | 0 | 10 |
| Col | 3 | 4 | 17 | 0 | 1 | 3 | 0.190476 | 0.19047619 | 0.142857 | 12 |
| Col | 3 | 5 | 8 | 0 | 2 | 1 | 0.272727 | 0.272727273 | 0.090909 | 12 |
| Col | 5 | 1 | 25 | 0 | 3 | 4 | 0.21875 | 0.21875 | 0.125 | 12 |
| Col | 1 | 3 | 19 | 0 | 1 | 5 | 0.24 | 0.24 | 0.2 | 13 |
| Col | 2 | 1 | 33 | 1 | 0 | 1 | 0.057143 | 0.028571429 | 0.028571 | 13 |
| Col | 2 | 4 | 16 | 0 | 3 | 1 | 0.2 | 0.2 | 0.05 | 13 |
| Col | 3 | 1 | 26 | 0 | 0 | 1 | 0.037037 | 0.037037037 | 0.037037 | 13 |
| Col | 3 | 4 | 26 | 1 | 2 | 2 | 0.16129 | 0.129032258 | 0.064516 | 13 |
| Col | 4 | 2 | 19 | 2 | 0 | 1 | 0.136364 | 0.045454545 | 0.045455 | 13 |
| Col | 4 | 5 | 11 | 0 | 1 | 3 | 0.266667 | 0.266666667 | 0.2 | 13 |
| Col | 5 | 2 | 19 | 0 | 1 | 5 | 0.24 | 0.24 | 0.2 | 13 |
| Col | 5 | 5 | 18 | 1 | 0 | 2 | 0.142857 | 0.095238095 | 0.095238 | 13 |
| Col | 6 | 3 | 29 | 0 | 4 | 1 | 0.147059 | 0.147058824 | 0.029412 | 13 |
| Col | 4 | 4 | 13 | 0 | 0 | 5 | 0.277778 | 0.277777778 | 0.277778 | 14 |
| Col | 5 | 1 | 23 | 0 | 1 | 3 | 0.148148 | 0.148148148 | 0.111111 | 14 |
| Col | 6 | 1 | 21 | 0 | 3 | 2 | 0.192308 | 0.192307692 | 0.076923 | 14 |
| Col | 6 | 5 | 27 | 0 | 1 | 3 | 0.129032 | 0.129032258 | 0.096774 | 14 |
| Col | 7 | 2 | 25 | 0 | 1 | 1 | 0.074074 | 0.074074074 | 0.037037 | 14 |
| Col | 8 | 2 | 17 | 2 | 2 | 6 | 0.37037 | 0.296296296 | 0.222222 | 14 |
| Col | 1 | 5 | 20 | 1 | 2 | 4 | 0.259259 | 0.222222222 | 0.148148 | 15 |
| Col | 2 | 4 | 21 | 0 | 0 | 7 | 0.25 | 0.25 | 0.25 | 15 |
| Col | 3 | 5 | 15 | 0 | 3 | 3 | 0.285714 | 0.285714286 | 0.142857 | 15 |
| Col | 4 | 4 | 21 | 3 | 4 | 3 | 0.322581 | 0.225806452 | 0.096774 | 15 |
| Col | 5 | 1 | 11 | 0 | 1 | 0 | 0.083333 | 0.083333333 | 0 | 15 |
| Col | 6 | 2 | 20 | 0 | 2 | 4 | 0.230769 | 0.230769231 | 0.153846 | 15 |
| Col | 7 | 1 | 18 | 0 | 0 | 2 | 0.1 | 0.1 | 0.1 | 15 |
| Col | 8 | 3 | 19 | 0 | 1 | 0 | 0.05 | 0.05 | 0 | 15 |
| Col | 1 | 3 | 22 | 3 | 0 | 0 | 0.12 | 0 | 0 | 16 |
| Col | 2 | 4 | 24 | 1 | 4 | 3 | 0.25 | 0.21875 | 0.09375 | 16 |
| Col | 2 | 5 | 22 | 2 | 10 | 2 | 0.388889 | 0.333333333 | 0.055556 | 16 |
| Col | 3 | 3 | 34 | 2 | 6 | 8 | 0.32 | 0.28 | 0.16 | 16 |
| Col | 4 | 5 | 40 | 2 | 6 | 3 | 0.215686 | 0.176470588 | 0.058824 | 16 |
| Col | 5 | 1 | 40 | 3 | 5 | 0 | 0.166667 | 0.104166667 | 0 | 16 |
| Col | 6 | 5 | 28 | 0 | 2 | 1 | 0.096774 | 0.096774194 | 0.032258 | 16 |
| Col | 1 | 2 | 30 | 1 | 0 | 2 | 0.090909 | 0.060606061 | 0.060606 | 17 |
| Col | 2 | 4 | 22 | 0 | 1 | 7 | 0.266667 | 0.266666667 | 0.233333 | 17 |
| Col | 2 | 5 | 22 | 2 | 2 | 0 | 0.153846 | 0.076923077 | 0 | 17 |
| Col | 3 | 4 | 20 | 1 | 2 | 3 | 0.230769 | 0.192307692 | 0.115385 | 17 |
| Col | 4 | 2 | 21 | 0 | 3 | 2 | 0.192308 | 0.192307692 | 0.076923 | 17 |
| Col | 5 | 4 | 34 | 0 | 4 | 2 | 0.15 | 0.15 | 0.05 | 17 |
| Col | 6 | 2 | 26 | 0 | 3 | 5 | 0.235294 | 0.235294118 | 0.147059 | 17 |
| Col | 1 | 1 | 12 | 1 | 3 | 3 | 0.368421 | 0.315789474 | 0.157895 | 18 |
| Col | 2 | 5 | 21 | 0 | 4 | 5 | 0.3 | 0.3 | 0.166667 | 18 |
| Col | 3 | 4 | 32 | 1 | 2 | 3 | 0.157895 | 0.131578947 | 0.078947 | 18 |
| Col | 4 | 2 | 22 | 0 | 1 | 2 | 0.12 | 0.12 | 0.08 | 18 |
| Col | 5 | 2 | 30 | 2 | 0 | 3 | 0.142857 | 0.085714286 | 0.085714 | 18 |
| Col | 6 | 3 | 20 | 1 | 0 | 6 | 0.259259 | 0.222222222 | 0.222222 | 18 |
| Col | 7 | 2 | 25 | 1 | 6 | 3 | 0.285714 | 0.257142857 | 0.085714 | 18 |
| Col | 8 | 5 | 13 | 0 | 5 | 9 | 0.518519 | 0.518518519 | 0.333333 | 18 |
| Col | 1 | 2 | 18 | 0 | 3 | 1 | 0.181818 | 0.181818182 | 0.045455 | 19 |
| Col | 2 | 1 | 32 | 2 | 8 | 0 | 0.238095 | 0.19047619 | 0 | 19 |
| Col | 3 | 3 | 20 | 0 | 4 | 4 | 0.285714 | 0.285714286 | 0.142857 | 19 |
| Col | 4 | 1 | 12 | 1 | 0 | 0 | 0.076923 | 0 | 0 | 19 |
| Col | 5 | 4 | 18 | 0 | 1 | 2 | 0.142857 | 0.142857143 | 0.095238 | 19 |
| Col | 6 | 3 | 18 | 1 | 0 | 0 | 0.052632 | 0 | 0 | 19 |
| Col | 7 | 4 | 30 | 0 | 0 | 3 | 0.090909 | 0.090909091 | 0.090909 | 19 |
| Col | 8 | 2 | 9 | 0 | 0 | 2 | 0.181818 | 0.181818182 | 0.181818 | 19 |
| Col | 1 | 2 | 24 | 0 | 3 | 5 | 0.25 | 0.25 | 0.15625 | 20 |
| Col | 2 | 5 | 33 | 0 | 2 | 2 | 0.108108 | 0.108108108 | 0.054054 | 20 |
| Col | 3 | 5 | 27 | 0 | 2 | 0 | 0.068966 | 0.068965517 | 0 | 20 |
| Col | 4 | 4 | 25 | 2 | 2 | 4 | 0.242424 | 0.181818182 | 0.121212 | 20 |
| Col | 5 | 3 | 16 | 0 | 2 | 1 | 0.157895 | 0.157894737 | 0.052632 | 20 |
| Col | 6 | 4 | 19 | 3 | 4 | 10 | 0.472222 | 0.388888889 | 0.277778 | 20 |
| Col | 7 | 3 | 18 | 1 | 0 | 4 | 0.217391 | 0.173913043 | 0.173913 | 20 |
| Col | 8 | 4 | 23 | 1 | 6 | 0 | 0.233333 | 0.2 | 0 | 20 |
| Col | 1 | 2 | 17 | 0 | 0 | 3 | 0.15 | 0.15 | 0.15 | 21 |
| Col | 2 | 1 | 15 | 1 | 0 | 0 | 0.0625 | 0 | 0 | 21 |
| Col | 3 | 3 | 30 | 0 | 3 | 3 | 0.166667 | 0.166666667 | 0.083333 | 21 |
| Col | 5 | 4 | 16 | 2 | 2 | 7 | 0.407407 | 0.333333333 | 0.259259 | 21 |
| Col | 6 | 1 | 23 | 1 | 3 | 1 | 0.178571 | 0.142857143 | 0.035714 | 21 |
| Col | 7 | 3 | 26 | 3 | 2 | 1 | 0.1875 | 0.09375 | 0.03125 | 21 |
| Col | 1 | 1 | 25 | 0 | 1 | 0 | 0.038462 | 0.038461538 | 0 | 22 |
| Col | 2 | 3 | 30 | 0 | 1 | 5 | 0.166667 | 0.166666667 | 0.138889 | 22 |
| Col | 4 | 1 | 22 | 0 | 5 | 4 | 0.290323 | 0.290322581 | 0.129032 | 22 |
| Col | 5 | 2 | 30 | 1 | 5 | 1 | 0.189189 | 0.162162162 | 0.027027 | 22 |
| Col | 6 | 1 | 33 | 2 | 3 | 2 | 0.175 | 0.125 | 0.05 | 22 |
| Col | 1 | 1 | 22 | 2 | 5 | 1 | 0.266667 | 0.2 | 0.033333 | 23 |
| Col | 2 | 5 | 21 | 1 | 1 | 0 | 0.086957 | 0.043478261 | 0 | 23 |
| Col | 3 | 3 | 12 | 3 | 0 | 1 | 0.25 | 0.0625 | 0.0625 | 23 |
| Col | 4 | 2 | 27 | 0 | 0 | 2 | 0.068966 | 0.068965517 | 0.068966 | 23 |
| Col | 5 | 3 | 18 | 1 | 5 | 3 | 0.333333 | 0.296296296 | 0.111111 | 23 |
| Col | 6 | 3 | 25 | 0 | 2 | 5 | 0.21875 | 0.21875 | 0.15625 | 23 |
| Col | 7 | 1 | 15 | 1 | 2 | 0 | 0.166667 | 0.111111111 | 0 | 23 |
| Col | 1 | 1 | 10 | 1 | 1 | 0 | 0.166667 | 0.083333333 | 0 | 25 |
| Col | 3 | 3 | 33 | 0 | 0 | 3 | 0.083333 | 0.083333333 | 0.083333 | 25 |
| Col | 5 | 3 | 20 | 0 | 2 | 3 | 0.2 | 0.2 | 0.12 | 25 |
| Col | 3 | 5 | 28 | 0 | 7 | 1 | 0.222222 | 0.222222222 | 0.027778 | 26 |
| Col | 4 | 2 | 35 | 0 | 5 | 0 | 0.125 | 0.125 | 0 | 26 |
| Col | 5 | 1 | 25 | 0 | 1 | 3 | 0.137931 | 0.137931034 | 0.103448 | 26 |
| Col | 7 | 5 | 22 | 1 | 1 | 4 | 0.214286 | 0.178571429 | 0.142857 | 26 |
| Col | 8 | 4 | 38 | 2 | 4 | 3 | 0.191489 | 0.14893617 | 0.06383 | 26 |
| Col | 2 | 1 | 18 | 0 | 1 | 3 | 0.181818 | 0.181818182 | 0.136364 | 27 |
| Col | 3 | 2 | 21 | 0 | 2 | 3 | 0.192308 | 0.192307692 | 0.115385 | 27 |
| Col | 5 | 1 | 20 | 0 | 1 | 6 | 0.259259 | 0.259259259 | 0.222222 | 27 |
| Col | 6 | 1 | 21 | 0 | 0 | 1 | 0.045455 | 0.045454545 | 0.045455 | 27 |
| Col | 7 | 3 | 14 | 0 | 2 | 2 | 0.222222 | 0.222222222 | 0.111111 | 27 |
| Col | 8 | 3 | 19 | 0 | 0 | 6 | 0.24 | 0.24 | 0.24 | 27 |
| Col | 1 | 2 | 25 | 0 | 1 | 3 | 0.137931 | 0.137931034 | 0.103448 | 28 |
| Col | 2 | 4 | 27 | 0 | 2 | 0 | 0.068966 | 0.068965517 | 0 | 28 |
| Col | 3 | 5 | 20 | 0 | 1 | 0 | 0.047619 | 0.047619048 | 0 | 28 |
| Col | 5 | 3 | 23 | 1 | 1 | 2 | 0.148148 | 0.111111111 | 0.074074 | 28 |
| Col | 6 | 2 | 21 | 0 | 2 | 5 | 0.25 | 0.25 | 0.178571 | 28 |
| Col | 7 | 5 | 16 | 4 | 5 | 2 | 0.407407 | 0.259259259 | 0.074074 | 28 |
| Col | 2 | 1 | 11 | 1 | 7 | 2 | 0.47619 | 0.428571429 | 0.095238 | 29 |
| Col | 5 | 2 | 13 | 0 | 1 | 2 | 0.1875 | 0.1875 | 0.125 | 29 |
| Col | 6 | 4 | 11 | 2 | 3 | 1 | 0.352941 | 0.235294118 | 0.058824 | 29 |
| Col | 7 | 2 | 19 | 1 | 2 | 0 | 0.136364 | 0.090909091 | 0 | 29 |
| Col | 1 | 3 | 43 | 1 | 1 | 3 | 0.104167 | 0.083333333 | 0.0625 | 31 |
| Col | 2 | 3 | 26 | 0 | 1 | 0 | 0.037037 | 0.037037037 | 0 | 31 |
| Col | 3 | 3 | 25 | 0 | 4 | 3 | 0.21875 | 0.21875 | 0.09375 | 31 |
| Col | 4 | 4 | 31 | 1 | 3 | 1 | 0.138889 | 0.111111111 | 0.027778 | 31 |
| Col | 6 | 5 | 31 | 0 | 3 | 0 | 0.088235 | 0.088235294 | 0 | 31 |
| Col | 1 | 3 | 19 | 1 | 2 | 1 | 0.173913 | 0.130434783 | 0.043478 | 32 |
| Col | 2 | 1 | 14 | 0 | 2 | 2 | 0.222222 | 0.222222222 | 0.111111 | 32 |
| Col | 5 | 2 | 20 | 1 | 2 | 1 | 0.166667 | 0.125 | 0.041667 | 32 |
| Col | 6 | 4 | 23 | 1 | 0 | 2 | 0.115385 | 0.076923077 | 0.076923 | 32 |
| Col | 3 | 5 | 24 | 0 | 7 | 3 | 0.294118 | 0.294117647 | 0.088235 | 33 |
| Col | 6 | 2 | 12 | 0 | 0 | 1 | 0.076923 | 0.076923077 | 0.076923 | 33 |
| Col | 7 | 3 | 25 | 0 | 2 | 4 | 0.193548 | 0.193548387 | 0.129032 | 33 |
| Col | 1 | 2 | 22 | 1 | 0 | 5 | 0.214286 | 0.178571429 | 0.178571 | 34 |
| Col | 2 | 1 | 33 | 1 | 7 | 0 | 0.195122 | 0.170731707 | 0 | 34 |
| Col | 3 | 5 | 24 | 2 | 2 | 0 | 0.142857 | 0.071428571 | 0 | 34 |
| Col | 4 | 1 | 21 | 1 | 0 | 0 | 0.045455 | 0 | 0 | 34 |
| Col | 6 | 1 | 25 | 1 | 0 | 0 | 0.038462 | 0 | 0 | 34 |
| Col | 7 | 5 | 11 | 1 | 0 | 4 | 0.3125 | 0.25 | 0.25 | 34 |
| Col | 8 | 1 | 22 | 2 | 0 | 0 | 0.083333 | 0 | 0 | 34 |
| Col | 1 | 4 | 26 | 0 | 3 | 11 | 0.35 | 0.35 | 0.275 | 35 |
| Col | 3 | 5 | 30 | 1 | 2 | 1 | 0.117647 | 0.088235294 | 0.029412 | 35 |
| Col | 4 | 2 | 24 | 0 | 9 | 2 | 0.314286 | 0.314285714 | 0.057143 | 35 |
| Col | 5 | 5 | 20 | 1 | 1 | 0 | 0.090909 | 0.045454545 | 0 | 35 |
| Col | 6 | 5 | 16 | 1 | 2 | 0 | 0.157895 | 0.105263158 | 0 | 35 |
| Col | 1 | 4 | 26 | 0 | 3 | 11 | 0.35 | 0.35 | 0.275 | 36 |
| Col | 3 | 5 | 30 | 1 | 2 | 1 | 0.117647 | 0.088235294 | 0.029412 | 36 |
| Col | 4 | 2 | 24 | 0 | 9 | 2 | 0.314286 | 0.314285714 | 0.057143 | 36 |
| Col | 5 | 5 | 20 | 1 | 1 | 0 | 0.090909 | 0.045454545 | 0 | 36 |
| Col | 6 | 5 | 16 | 1 | 2 | 0 | 0.157895 | 0.105263158 | 0 | 36 |
| Col | 2 | 4 | 19 | 2 | 2 | 3 | 0.269231 | 0.192307692 | 0.115385 | 37 |
| Col | 3 | 4 | 20 | 1 | 2 | 2 | 0.2 | 0.16 | 0.08 | 37 |
| Col | 6 | 3 | 12 | 1 | 6 | 3 | 0.454545 | 0.409090909 | 0.136364 | 37 |
| Col | 7 | 2 | 15 | 0 | 0 | 1 | 0.0625 | 0.0625 | 0.0625 | 37 |
| Col | 8 | 2 | 20 | 1 | 1 | 0 | 0.090909 | 0.045454545 | 0 | 37 |
| Col | 2 | 3 | 13 | 0 | 1 | 2 | 0.1875 | 0.1875 | 0.125 | 1 |
| Col | 3 | 5 | 8 | 0 | 2 | 0 | 0.2 | 0.2 | 0 | 1 |
| Col | 5 | 4 | 15 | 1 | 3 | 1 | 0.25 | 0.2 | 0.05 | 1 |
| Col | 1 | 3 | 29 | 0 | 0 | 1 | 0.033333 | 0.033333333 | 0.033333 | 40 |
| Col | 2 | 1 | 24 | 0 | 1 | 0 | 0.04 | 0.04 | 0 | 40 |
| Col | 3 | 3 | 20 | 0 | 2 | 0 | 0.090909 | 0.090909091 | 0 | 40 |
| Col | 1 | 1 | 19 | 2 | 10 | 5 | 0.472222 | 0.416666667 | 0.138889 | 41 |
| Col | 2 | 1 | 17 | 1 | 2 | 3 | 0.26087 | 0.217391304 | 0.130435 | 41 |
| Col | 3 | 2 | 26 | 0 | 1 | 2 | 0.103448 | 0.103448276 | 0.068966 | 41 |
| Col | 5 | 4 | 14 | 0 | 0 | 2 | 0.125 | 0.125 | 0.125 | 41 |
| Col | 6 | 4 | 16 | 0 | 1 | 1 | 0.111111 | 0.111111111 | 0.055556 | 41 |
| Col | 3 | 2 | 33 | 1 | 1 | 0 | 0.057143 | 0.028571429 | 0 | 42 |
| Col | 5 | 2 | 14 | 1 | 2 | 1 | 0.222222 | 0.166666667 | 0.055556 | 42 |
| Col | 1 | 2 | 17 | 0 | 1 | 0 | 0.055556 | 0.055555556 | 0 | 43 |
| Col | 3 | 3 | 22 | 0 | 0 | 2 | 0.083333 | 0.083333333 | 0.083333 | 43 |
| Col | 4 | 2 | 14 | 1 | 1 | 2 | 0.222222 | 0.166666667 | 0.111111 | 43 |
| Col | 1 | 5 | 30 | 1 | 1 | 3 | 0.142857 | 0.114285714 | 0.085714 | 44 |
| Col | 2 | 3 | 25 | 0 | 0 | 1 | 0.038462 | 0.038461538 | 0.038462 | 44 |
| Col | 6 | 3 | 35 | 1 | 2 | 3 | 0.146341 | 0.12195122 | 0.073171 | 44 |
| Col | 1 | 1 | 19 | 0 | 1 | 1 | 0.095238 | 0.095238095 | 0.047619 | 45 |
| Col | 2 | 3 | 15 | 1 | 1 | 3 | 0.25 | 0.2 | 0.15 | 45 |
| Col | 4 | 5 | 10 | 1 | 1 | 4 | 0.375 | 0.3125 | 0.25 | 45 |
| *cpr5* | 3 | 5 | 22 | 0 | 1 | 1 | 0.083333 | 0.083333333 | 0.041667 | 30 |
| *cpr5* | 4 | 5 | 26 | 0 | 1 | 1 | 0.071429 | 0.071428571 | 0.035714 | 30 |
| *cpr5* | 5 | 5 | 28 | 0 | 1 | 2 | 0.096774 | 0.096774194 | 0.064516 | 30 |
| *cpr5* | 6 | 2 | 10 | 0 | 0 | 0 | 0 | 0 | 0 | 30 |
| *cpr5* | 7 | 3 | 23 | 0 | 0 | 0 | 0 | 0 | 0 | 30 |
| *cpr5* | 8 | 4 | 18 | 0 | 1 | 1 | 0.1 | 0.1 | 0.05 | 30 |
| *cpr5* | 1 | 5 | 34 | 0 | 1 | 1 | 0.055556 | 0.055555556 | 0.027778 | 31 |
| *cpr5* | 3 | 1 | 21 | 0 | 2 | 0 | 0.086957 | 0.086956522 | 0 | 31 |
| *cpr5* | 4 | 2 | 20 | 0 | 0 | 0 | 0 | 0 | 0 | 31 |
| *cpr5* | 5 | 2 | 12 | 0 | 0 | 0 | 0 | 0 | 0 | 31 |
| *cpr5* | 6 | 3 | 18 | 0 | 0 | 1 | 0.052632 | 0.052631579 | 0.052632 | 31 |
| *cpr5* | 1 | 2 | 26 | 0 | 0 | 2 | 0.071429 | 0.071428571 | 0.071429 | 32 |
| *cpr5* | 2 | 5 | 26 | 0 | 2 | 3 | 0.16129 | 0.161290323 | 0.096774 | 32 |
| *cpr5* | 3 | 2 | 11 | 0 | 0 | 0 | 0 | 0 | 0 | 32 |
| *cpr5* | 6 | 2 | 24 | 3 | 1 | 1 | 0.172414 | 0.068965517 | 0.034483 | 32 |
| *csn5* | 1 | 5 | 21 | 0 | 0 | 1 | 0.045455 | 0.045454545 | 0.045455 | 27 |
| *csn5* | 3 | 3 | 22 | 0 | 3 | 2 | 0.185185 | 0.185185185 | 0.074074 | 27 |
| *csn5* | 2 | 4 | 30 | 2 | 2 | 4 | 0.210526 | 0.157894737 | 0.105263 | 27 |
| *csn5* | 4 | 2 | 24 | 1 | 0 | 4 | 0.172414 | 0.137931034 | 0.137931 | 27 |
| *csn5* | 5 | 5 | 31 | 0 | 4 | 3 | 0.184211 | 0.184210526 | 0.078947 | 27 |
| *csn5* | 6 | 3 | 33 | 4 | 5 | 4 | 0.282609 | 0.195652174 | 0.086957 | 27 |
| *csn5* | 7 | 2 | 28 | 1 | 4 | 2 | 0.2 | 0.171428571 | 0.057143 | 27 |
| *csn5* | 8 | 2 | 28 | 0 | 4 | 1 | 0.151515 | 0.151515152 | 0.030303 | 27 |
| *csn5* | 1 | 1 | 26 | 0 | 1 | 1 | 0.071429 | 0.071428571 | 0.035714 | 30 |
| *csn5* | 2 | 4 | 25 | 0 | 1 | 2 | 0.107143 | 0.107142857 | 0.071429 | 30 |
| *csn5* | 3 | 4 | 24 | 0 | 1 | 3 | 0.142857 | 0.142857143 | 0.107143 | 30 |
| *csn5* | 4 | 4 | 28 | 0 | 1 | 4 | 0.151515 | 0.151515152 | 0.121212 | 30 |
| *csn5* | 5 | 3 | 27 | 0 | 0 | 1 | 0.035714 | 0.035714286 | 0.035714 | 30 |
| *csn5* | 6 | 5 | 29 | 2 | 1 | 2 | 0.147059 | 0.088235294 | 0.058824 | 30 |
| *csn5* | 7 | 5 | 35 | 2 | 3 | 5 | 0.222222 | 0.177777778 | 0.111111 | 30 |
| *csn5* | 8 | 5 | 26 | 0 | 5 | 2 | 0.212121 | 0.212121212 | 0.060606 | 30 |
| *dde2* | 1 | 1 | 48 | 0 | 2 | 1 | 0.058824 | 0.058823529 | 0.019608 | 11 |
| *dde2* | 3 | 2 | 26 | 0 | 0 | 0 | 0 | 0 | 0 | 11 |
| *dde2* | 5 | 3 | 28 | 0 | 0 | 0 | 0 | 0 | 0 | 11 |
| *dde2* | 6 | 4 | 25 | 0 | 0 | 4 | 0.137931 | 0.137931034 | 0.137931 | 11 |
| *dde2* | 2 | 3 | 18 | 0 | 1 | 2 | 0.142857 | 0.142857143 | 0.095238 | 29 |
| *dde2* | 4 | 4 | 25 | 1 | 1 | 1 | 0.107143 | 0.071428571 | 0.035714 | 29 |
| *dde2* | 5 | 3 | 16 | 1 | 0 | 3 | 0.2 | 0.15 | 0.15 | 29 |
| *dde2* | 5 | 5 | 22 | 1 | 4 | 0 | 0.185185 | 0.148148148 | 0 | 29 |
| *dde2* | 6 | 2 | 17 | 0 | 5 | 2 | 0.291667 | 0.291666667 | 0.083333 | 29 |
| *dde2* | 7 | 3 | 30 | 1 | 2 | 0 | 0.090909 | 0.060606061 | 0 | 29 |
| *dde2* | 1 | 4 | 22 | 1 | 0 | 1 | 0.083333 | 0.041666667 | 0.041667 | 44 |
| *dde2* | 3 | 2 | 48 | 1 | 3 | 0 | 0.076923 | 0.057692308 | 0 | 44 |
| *dde2* | 4 | 5 | 20 | 0 | 1 | 1 | 0.090909 | 0.090909091 | 0.045455 | 44 |
| *dde2* | 6 | 4 | 22 | 1 | 0 | 0 | 0.043478 | 0 | 0 | 44 |
| *dde2* | 7 | 3 | 19 | 1 | 0 | 0 | 0.05 | 0 | 0 | 44 |
| *dde2/pad4* | 1 | 1 | 32 | 0 | 0 | 2 | 0.058824 | 0.058823529 | 0.058824 | 14 |
| *dde2/pad4* | 2 | 4 | 29 | 0 | 1 | 4 | 0.147059 | 0.147058824 | 0.117647 | 14 |
| *dde2/pad4* | 3 | 1 | 20 | 1 | 0 | 1 | 0.090909 | 0.045454545 | 0.045455 | 14 |
| *dde2/pad4* | 3 | 4 | 24 | 0 | 2 | 3 | 0.172414 | 0.172413793 | 0.103448 | 14 |
| *dde2/pad4* | 4 | 5 | 17 | 0 | 0 | 2 | 0.105263 | 0.105263158 | 0.105263 | 14 |
| *dde2/pad4* | 5 | 2 | 37 | 0 | 0 | 5 | 0.119048 | 0.119047619 | 0.119048 | 14 |
| *dde2/pad4* | 6 | 3 | 24 | 0 | 0 | 3 | 0.111111 | 0.111111111 | 0.111111 | 14 |
| *dde2/pad4* | 7 | 3 | 30 | 1 | 2 | 1 | 0.117647 | 0.088235294 | 0.029412 | 14 |
| *dde2/pad4* | 8 | 3 | 22 | 0 | 0 | 1 | 0.043478 | 0.043478261 | 0.043478 | 14 |
| *dde2/pad4* | 1 | 1 | 16 | 0 | 0 | 0 | 0 | 0 | 0 | 15 |
| *dde2/pad4* | 2 | 5 | 18 | 0 | 3 | 0 | 0.142857 | 0.142857143 | 0 | 15 |
| *dde2/pad4* | 3 | 2 | 19 | 1 | 1 | 3 | 0.208333 | 0.166666667 | 0.125 | 15 |
| *dde2/pad4* | 4 | 5 | 19 | 1 | 0 | 1 | 0.095238 | 0.047619048 | 0.047619 | 15 |
| *dde2/pad4* | 5 | 5 | 20 | 4 | 3 | 1 | 0.285714 | 0.142857143 | 0.035714 | 15 |
| *dde2/pad4* | 6 | 3 | 25 | 0 | 0 | 1 | 0.038462 | 0.038461538 | 0.038462 | 15 |
| *dde2/pad4* | 7 | 2 | 24 | 0 | 2 | 0 | 0.076923 | 0.076923077 | 0 | 15 |
| *dde2/pad4* | 8 | 2 | 23 | 2 | 0 | 0 | 0.08 | 0 | 0 | 15 |
| *dde2/pad4* | 1 | 4 | 19 | 1 | 2 | 1 | 0.173913 | 0.130434783 | 0.043478 | 29 |
| *dde2/pad4* | 2 | 4 | 31 | 1 | 2 | 0 | 0.088235 | 0.058823529 | 0 | 29 |
| *dde2/pad4* | 3 | 3 | 11 | 0 | 0 | 1 | 0.083333 | 0.083333333 | 0.083333 | 29 |
| *dde2/pad4* | 4 | 3 | 33 | 2 | 2 | 1 | 0.131579 | 0.078947368 | 0.026316 | 29 |
| *dde2/pad4* | 6 | 5 | 11 | 0 | 1 | 0 | 0.083333 | 0.083333333 | 0 | 29 |
| *dde2/pad4* | 7 | 4 | 25 | 1 | 0 | 1 | 0.074074 | 0.037037037 | 0.037037 | 29 |
| *dde2/sid2* | 1 | 3 | 20 | 0 | 2 | 1 | 0.130435 | 0.130434783 | 0.043478 | 29 |
| *dde2/sid2* | 2 | 2 | 22 | 0 | 2 | 2 | 0.153846 | 0.153846154 | 0.076923 | 29 |
| *dde2/sid2* | 3 | 2 | 16 | 0 | 1 | 2 | 0.157895 | 0.157894737 | 0.105263 | 29 |
| *dde2/sid2* | 3 | 4 | 7 | 0 | 0 | 1 | 0.125 | 0.125 | 0.125 | 29 |
| *dde2/sid2* | 4 | 1 | 17 | 1 | 0 | 0 | 0.055556 | 0 | 0 | 29 |
| *dde2/sid2* | 4 | 2 | 21 | 0 | 1 | 2 | 0.125 | 0.125 | 0.083333 | 29 |
| *dde2/sid2* | 5 | 4 | 29 | 1 | 3 | 2 | 0.171429 | 0.142857143 | 0.057143 | 29 |
| *dde2/sid2* | 6 | 3 | 21 | 1 | 5 | 2 | 0.275862 | 0.24137931 | 0.068966 | 29 |
| *dde2/sid2* | 7 | 5 | 18 | 3 | 0 | 2 | 0.217391 | 0.086956522 | 0.086957 | 29 |
| *dde2/sid2* | 1 | 2 | 5 | 0 | 0 | 0 | 0 | 0 | 0 | 12 |
| *dde2/sid2* | 2 | 1 | 17 | 0 | 1 | 1 | 0.105263 | 0.105263158 | 0.052632 | 12 |
| *dde2/sid2* | 2 | 4 | 6 | 0 | 0 | 0 | 0 | 0 | 0 | 12 |
| *dde2/sid2* | 3 | 3 | 16 | 0 | 0 | 2 | 0.111111 | 0.111111111 | 0.111111 | 12 |
| *dde2/sid2* | 5 | 4 | 45 | 0 | 1 | 1 | 0.042553 | 0.042553191 | 0.021277 | 12 |
| *dde2/sid2* | 7 | 4 | 10 | 0 | 0 | 0 | 0 | 0 | 0 | 12 |
| *Della quant mutant* |  |  | 32 | 0 | 0 | 1 | 0.030303 | 0.03030303 | 0.030303 | 4 |
| *Della quant mutant* |  |  | 20 | 1 | 2 | 3 | 0.230769 | 0.192307692 | 0.115385 | 4 |
| *Della quant mutant* |  |  | 33 | 0 | 5 | 1 | 0.153846 | 0.153846154 | 0.025641 | 4 |
| *Della quant mutant* |  |  | 22 | 0 | 6 | 4 | 0.3125 | 0.3125 | 0.125 | 4 |
| *Della quant mutant* |  |  | 37 | 0 | 2 | 0 | 0.051282 | 0.051282051 | 0 | 4 |
| *Della quant mutant* |  |  | 25 | 2 | 5 | 1 | 0.242424 | 0.181818182 | 0.030303 | 4 |
| *Della quant mutant* | 1 | 1 | 23 | 0 | 2 | 0 | 0.08 | 0.08 | 0 | 7 |
| *Della quant mutant* | 2 | 2 | 24 | 0 | 1 | 0 | 0.04 | 0.04 | 0 | 7 |
| *Della quant mutant* | 4 | 1 | 31 | 0 | 5 | 0 | 0.138889 | 0.138888889 | 0 | 7 |
| *Della quant mutant* | 5 | 4 | 21 | 0 | 0 | 0 | 0 | 0 | 0 | 7 |
| *Della quant mutant* | 6 | 3 | 32 | 2 | 6 | 0 | 0.2 | 0.15 | 0 | 7 |
| *Della quant mutant* | 1 | 3 | 17 | 0 | 0 | 0 | 0 | 0 | 0 | 1 |
| *Della quant mutant* | 3 | 1 | 21 | 0 | 2 | 0 | 0.086957 | 0.086956522 | 0 | 1 |
| *Della quant mutant* | 4 | 3 | 19 | 1 | 1 | 0 | 0.095238 | 0.047619048 | 0 | 1 |
| *edr1* | 2 | 1 | 9 | 0 | 0 | 3 | 0.25 | 0.25 | 0.25 | 45 |
| *edr1* | 4 | 1 | 14 | 0 | 0 | 4 | 0.222222 | 0.222222222 | 0.222222 | 45 |
| *edr1* | 1 | 3 | 15 | 2 | 0 | 0 | 0.117647 | 0 | 0 | 41 |
| *edr1* | 3 | 5 | 25 | 0 | 0 | 1 | 0.038462 | 0.038461538 | 0.038462 | 41 |
| *edr1* | 4 | 3 | 7 | 0 | 1 | 0 | 0.125 | 0.125 | 0 | 41 |
| *edr1* | 5 | 3 | 14 | 0 | 1 | 2 | 0.176471 | 0.176470588 | 0.117647 | 41 |
| *edr1* | 8 | 3 | 12 | 0 | 0 | 1 | 0.076923 | 0.076923077 | 0.076923 | 41 |
| *edr1* | 2 | 4 | 15 | 0 | 0 | 0 | 0 | 0 | 0 | 43 |
| *edr1* | 6 | 2 | 19 | 1 | 1 | 5 | 0.269231 | 0.230769231 | 0.192308 | 43 |
| *edr1* | 8 | 4 | 21 | 0 | 0 | 1 | 0.045455 | 0.045454545 | 0.045455 | 43 |
| *edr1* | 5 | 2 | 22 | 0 | 0 | 0 | 0 | 0 | 0 | 44 |
| *edr1* | 7 | 4 | 14 | 1 | 0 | 0 | 0.066667 | 0 | 0 | 44 |
| *ein2-1* | 1 | 5 | 14 | 0 | 2 | 6 | 0.363636 | 0.363636364 | 0.272727 | 20 |
| *ein2-1* | 2 | 2 | 13 | 0 | 0 | 4 | 0.235294 | 0.235294118 | 0.235294 | 20 |
| *ein2-1* | 4 | 3 | 19 | 1 | 3 | 4 | 0.296296 | 0.259259259 | 0.148148 | 20 |
| *ein2-1* | 5 | 5 | 18 | 0 | 3 | 1 | 0.181818 | 0.181818182 | 0.045455 | 20 |
| *ein2-1* | 6 | 1 | 28 | 1 | 2 | 3 | 0.176471 | 0.147058824 | 0.088235 | 20 |
| *ein2-1* | 7 | 1 | 7 | 1 | 0 | 0 | 0.125 | 0 | 0 | 20 |
| *ein2-1* | 8 | 1 | 26 | 1 | 3 | 2 | 0.1875 | 0.15625 | 0.0625 | 20 |
| *ein2-1* | 1 | 4 | 27 | 2 | 1 | 6 | 0.25 | 0.194444444 | 0.166667 | 23 |
| *ein2-1* | 2 | 3 | 27 | 0 | 3 | 0 | 0.1 | 0.1 | 0 | 23 |
| *ein2-1* | 3 | 2 | 40 | 4 | 2 | 2 | 0.166667 | 0.083333333 | 0.041667 | 23 |
| *ein2-1* | 4 | 5 | 21 | 1 | 4 | 6 | 0.34375 | 0.3125 | 0.1875 | 23 |
| *ein2-1* | 6 | 2 | 19 | 1 | 2 | 2 | 0.208333 | 0.166666667 | 0.083333 | 23 |
| *ein2-1* | 7 | 5 | 24 | 1 | 0 | 3 | 0.142857 | 0.107142857 | 0.107143 | 23 |
| *ein2-1* | 1 | 4 | 24 | 0 | 0 | 2 | 0.076923 | 0.076923077 | 0.076923 | 26 |
| *ein2-1* | 7 | 1 | 23 | 0 | 1 | 4 | 0.178571 | 0.178571429 | 0.142857 | 26 |
| *ein2-1* | 7 | 2 | 46 | 1 | 3 | 8 | 0.206897 | 0.189655172 | 0.137931 | 26 |
| *jar1* | 1 | 2 | 19 | 0 | 0 | 0 | 0 | 0 | 0 | 8 |
| *jar1* | 3 | 5 | 10 | 0 | 0 | 0 | 0 | 0 | 0 | 8 |
| *jar1* | 4 | 3 | 22 | 1 | 3 | 1 | 0.185185 | 0.148148148 | 0.037037 | 8 |
| *jar1* | 5 | 1 | 19 | 0 | 2 | 2 | 0.173913 | 0.173913043 | 0.086957 | 8 |
| *jar1* | 6 | 4 | 17 | 1 | 3 | 1 | 0.227273 | 0.181818182 | 0.045455 | 8 |
| *jar1* | 7 | 1 | 16 | 0 | 2 | 0 | 0.111111 | 0.111111111 | 0 | 8 |
| *jar1* | 2 | 2 | 21 | 0 | 0 | 0 | 0 | 0 | 0 | 26 |
| *jar1* | 3 | 2 | 18 | 0 | 2 | 5 | 0.28 | 0.28 | 0.2 | 26 |
| *jar1* | 5 | 4 | 30 | 0 | 0 | 2 | 0.0625 | 0.0625 | 0.0625 | 26 |
| *jar1* | 1 | 2 | 23 | 0 | 0 | 1 | 0.041667 | 0.041666667 | 0.041667 | 30 |
| *jar1* | 2 | 1 | 23 | 0 | 0 | 0 | 0 | 0 | 0 | 30 |
| *jar1* | 5 | 2 | 27 | 0 | 5 | 3 | 0.228571 | 0.228571429 | 0.085714 | 30 |
| *jar1* | 6 | 1 | 18 | 1 | 5 | 0 | 0.25 | 0.208333333 | 0 | 30 |
| *jar1* | 7 | 4 | 28 | 1 | 5 | 3 | 0.243243 | 0.216216216 | 0.081081 | 30 |
| *jar1* | 1 | 4 | 29 | 0 | 2 | 0 | 0.064516 | 0.064516129 | 0 | 32 |
| *jar1* | 3 | 4 | 28 | 0 | 2 | 2 | 0.125 | 0.125 | 0.0625 | 32 |
| *jar1* | 6 | 5 | 27 | 1 | 3 | 0 | 0.129032 | 0.096774194 | 0 | 32 |
| *jar1/axr1* | 1 | 3 | 27 | 1 | 0 | 0 | 0.035714 | 0 | 0 | 34 |
| *jar1/axr1* | 2 | 3 | 21 | 0 | 1 | 0 | 0.045455 | 0.045454545 | 0 | 34 |
| *jar1/axr1* | 3 | 3 | 21 | 0 | 0 | 1 | 0.045455 | 0.045454545 | 0.045455 | 34 |
| *jar1/axr1* | 4 | 4 | 20 | 0 | 0 | 1 | 0.047619 | 0.047619048 | 0.047619 | 34 |
| *jar1/axr1* | 5 | 3 | 31 | 0 | 2 | 1 | 0.088235 | 0.088235294 | 0.029412 | 34 |
| *jar1/axr1* | 6 | 2 | 18 | 0 | 0 | 0 | 0 | 0 | 0 | 34 |
| *jar1/axr1* | 7 | 4 | 11 | 0 | 1 | 5 | 0.352941 | 0.352941176 | 0.294118 | 34 |
| *jar1/axr1* | 1 | 5 | 18 | 0 | 1 | 1 | 0.1 | 0.1 | 0.05 | 41 |
| *jar1/axr1* | 5 | 5 | 14 | 1 | 0 | 0 | 0.066667 | 0 | 0 | 41 |
| *jar1/axr1* | 6 | 1 | 22 | 0 | 0 | 1 | 0.043478 | 0.043478261 | 0.043478 | 41 |
| *jar1/axr1* | 3 | 1 | 26 | 2 | 0 | 0 | 0.071429 | 0 | 0 | 41 |
| *jar1/axr1* | 4 | 2 | 28 | 0 | 2 | 0 | 0.066667 | 0.066666667 | 0 | 41 |
| *jar1/mlo* | 1 | 5 | 12 | 0 | 0 | 4 | 0.25 | 0.25 | 0.25 | 13 |
| *jar1/mlo* | 1 | 3 | 11 | 0 | 2 | 2 | 0.266667 | 0.266666667 | 0.133333 | 13 |
| *jar1/mlo* | 4 | 4 | 21 | 0 | 1 | 1 | 0.086957 | 0.086956522 | 0.043478 | 13 |
| *jar1/mlo* | 5 | 1 | 10 | 2 | 0 | 0 | 0.166667 | 0 | 0 | 13 |
| *jar1/mlo* | 5 | 4 | 15 | 0 | 0 | 2 | 0.117647 | 0.117647059 | 0.117647 | 13 |
| *jar1/mlo* | 6 | 2 | 23 | 1 | 1 | 1 | 0.115385 | 0.076923077 | 0.038462 | 13 |
| *jar1/mlo* | 6 | 5 | 25 | 1 | 1 | 6 | 0.242424 | 0.212121212 | 0.181818 | 13 |
| *jar1/mlo* | 4 | 2 | 17 | 0 | 4 | 0 | 0.190476 | 0.19047619 | 0 | 33 |
| *jar1/mlo* | 5 | 4 | 19 | 0 | 0 | 0 | 0 | 0 | 0 | 33 |
| *jar1/mlo* | 7 | 4 | 25 | 0 | 1 | 2 | 0.107143 | 0.107142857 | 0.071429 | 33 |
| *jar1/mlo* | 3 | 3 | 22 | 0 | 2 | 0 | 0.083333 | 0.083333333 | 0 | 35 |
| *jar1/mlo* | 6 | 2 | 26 | 1 | 2 | 2 | 0.16129 | 0.129032258 | 0.064516 | 35 |
| *jar1/mlo* | 7 | 1 | 30 | 0 | 0 | 3 | 0.090909 | 0.090909091 | 0.090909 | 35 |
| *jar1/mlo* | 7 | 3 | 21 | 0 | 1 | 0 | 0.045455 | 0.045454545 | 0 | 35 |
| *jar1/mlo* | 3 | 3 | 22 | 0 | 2 | 0 | 0.083333 | 0.083333333 | 0 | 36 |
| *jar1/mlo* | 6 | 2 | 26 | 1 | 2 | 2 | 0.16129 | 0.129032258 | 0.064516 | 36 |
| *jar1/mlo* | 7 | 1 | 30 | 0 | 0 | 3 | 0.090909 | 0.090909091 | 0.090909 | 36 |
| *jar1/mlo* | 7 | 3 | 21 | 0 | 1 | 0 | 0.045455 | 0.045454545 | 0 | 36 |
| *JAZ3:T3* | 2 | 3 | 14 | 1 | 0 | 2 | 0.176471 | 0.117647059 | 0.117647 | 42 |
| *JAZ3:T3* | 4 | 2 | 25 | 1 | 1 | 4 | 0.193548 | 0.161290323 | 0.129032 | 42 |
| *JAZ3:T3* | 5 | 5 | 26 | 0 | 3 | 4 | 0.212121 | 0.212121212 | 0.121212 | 42 |
| *JAZ3:T3* | 1 | 1 | 18 | 1 | 3 | 2 | 0.25 | 0.208333333 | 0.083333 | 34 |
| *JAZ3:T3* | 3 | 2 | 13 | 0 | 0 | 3 | 0.1875 | 0.1875 | 0.1875 | 34 |
| *JAZ3:T3* | 4 | 5 | 26 | 0 | 0 | 4 | 0.133333 | 0.133333333 | 0.133333 | 34 |
| *JAZ3:T3* | 5 | 4 | 31 | 0 | 0 | 1 | 0.03125 | 0.03125 | 0.03125 | 34 |
| *JAZ3:T3* | 6 | 3 | 19 | 0 | 2 | 3 | 0.208333 | 0.208333333 | 0.125 | 34 |
| *JAZ3:T3* | 7 | 3 | 18 | 0 | 1 | 1 | 0.1 | 0.1 | 0.05 | 34 |
| *JAZ3:T3* | 8 | 5 | 23 | 0 | 0 | 2 | 0.08 | 0.08 | 0.08 | 34 |
| *JAZ3:T3* | 1 | 3 | 27 | 0 | 0 | 4 | 0.129032 | 0.129032258 | 0.129032 | 37 |
| *JAZ3:T3* | 2 | 5 | 28 | 0 | 5 | 4 | 0.243243 | 0.243243243 | 0.108108 | 37 |
| *JAZ3:T3* | 3 | 2 | 18 | 0 | 2 | 2 | 0.181818 | 0.181818182 | 0.090909 | 37 |
| *JAZ3:T3* | 5 | 4 | 26 | 0 | 1 | 1 | 0.071429 | 0.071428571 | 0.035714 | 37 |
| *JAZ3:T3* | 6 | 1 | 19 | 2 | 1 | 1 | 0.173913 | 0.086956522 | 0.043478 | 37 |
| *JAZ3:T3* | 7 | 3 | 21 | 1 | 1 | 2 | 0.16 | 0.12 | 0.08 | 37 |
| *jaz4/5/9* | 1 | 2 | 24 | 0 | 1 | 2 | 0.111111 | 0.111111111 | 0.074074 | 3 |
| *jaz4/5/9* | 1 | 5 | 31 | 1 | 1 | 0 | 0.060606 | 0.03030303 | 0 | 3 |
| *jaz4/5/9* | 2 | 2 | 22 | 1 | 0 | 1 | 0.083333 | 0.041666667 | 0.041667 | 3 |
| *jaz4/5/9* | 3 | 2 | 23 | 4 | 1 | 0 | 0.178571 | 0.035714286 | 0 | 3 |
| *jaz4/5/9* | 6 | 5 | 24 | 2 | 1 | 1 | 0.142857 | 0.071428571 | 0.035714 | 3 |
| *jaz4/5/9* | 1 | 3 | 28 | 0 | 0 | 1 | 0.034483 | 0.034482759 | 0.034483 | 7 |
| *jaz4/5/9* | 2 | 1 | 20 | 1 | 3 | 0 | 0.166667 | 0.125 | 0 | 7 |
| *jaz4/5/9* | 3 | 5 | 20 | 0 | 2 | 0 | 0.090909 | 0.090909091 | 0 | 7 |
| *jaz4/5/9* | 4 | 3 | 27 | 2 | 1 | 1 | 0.129032 | 0.064516129 | 0.032258 | 7 |
| *jaz4/5/9* | 5 | 2 | 29 | 0 | 1 | 0 | 0.033333 | 0.033333333 | 0 | 7 |
| *jaz4/5/9* | 6 | 1 | 31 | 1 | 8 | 0 | 0.225 | 0.2 | 0 | 7 |
| *jaz4/5/9* | 1 | 5 | 28 | 1 | 2 | 3 | 0.176471 | 0.147058824 | 0.088235 | 33 |
| *jaz4/5/9* | 2 | 4 | 11 | 1 | 3 | 1 | 0.3125 | 0.25 | 0.0625 | 33 |
| *jaz4/5/9* | 3 | 1 | 18 | 0 | 5 | 1 | 0.25 | 0.25 | 0.041667 | 33 |
| *jaz4/5/9* | 5 | 3 | 22 | 0 | 1 | 2 | 0.12 | 0.12 | 0.08 | 33 |
| *jaz4/5/9* | 6 | 1 | 29 | 0 | 0 | 1 | 0.033333 | 0.033333333 | 0.033333 | 33 |
| *jin1-1* | 2 | 4 | 29 | 0 | 2 | 2 | 0.121212 | 0.121212121 | 0.060606 | 2 |
| *jin1-1* | 3 | 2 | 30 | 0 | 3 | 4 | 0.189189 | 0.189189189 | 0.108108 | 2 |
| *jin1-1* | 4 | 5 | 25 | 1 | 0 | 3 | 0.137931 | 0.103448276 | 0.103448 | 2 |
| *jin1-1* | 6 | 2 | 20 | 0 | 1 | 1 | 0.090909 | 0.090909091 | 0.045455 | 2 |
| *jin1-1* | 1 | 5 | 27 | 0 | 7 | 2 | 0.25 | 0.25 | 0.055556 | 11 |
| *jin1-1* | 2 | 5 | 26 | 0 | 5 | 2 | 0.212121 | 0.212121212 | 0.060606 | 11 |
| *jin1-1* | 3 | 1 | 18 | 0 | 0 | 0 | 0 | 0 | 0 | 11 |
| *jin1-1* | 4 | 3 | 38 | 0 | 0 | 0 | 0 | 0 | 0 | 11 |
| *jin1-1* | 5 | 5 | 37 | 0 | 2 | 0 | 0.051282 | 0.051282051 | 0 | 11 |
| *jin1-1* | 6 | 3 | 37 | 0 | 5 | 2 | 0.159091 | 0.159090909 | 0.045455 | 11 |
| *jin1-1* | 1 | 3 | 19 | 3 | 2 | 3 | 0.296296 | 0.185185185 | 0.111111 | 17 |
| *jin1-1* | 2 | 3 | 14 | 0 | 5 | 0 | 0.263158 | 0.263157895 | 0 | 17 |
| *jin1-1* | 4 | 4 | 25 | 0 | 2 | 0 | 0.074074 | 0.074074074 | 0 | 17 |
| *jin1-1* | 5 | 3 | 22 | 2 | 2 | 1 | 0.185185 | 0.111111111 | 0.037037 | 17 |
| *jin1-1* | 8 | 2 | 26 | 1 | 3 | 1 | 0.16129 | 0.129032258 | 0.032258 | 17 |
| *jin1-1* | 2 | 5 | 25 | 0 | 0 | 2 | 0.074074 | 0.074074074 | 0.074074 | 28 |
| *jin1-1* | 3 | 4 | 37 | 0 | 1 | 2 | 0.075 | 0.075 | 0.05 | 28 |
| *jin1-1* | 4 | 3 | 29 | 0 | 4 | 4 | 0.216216 | 0.216216216 | 0.108108 | 28 |
| *jin1-1* | 5 | 4 | 41 | 1 | 2 | 4 | 0.145833 | 0.125 | 0.083333 | 28 |
| *jin1-1* | 7 | 1 | 18 | 2 | 4 | 1 | 0.28 | 0.2 | 0.04 | 28 |
| *lsd1-2* | 3 | 4 | 21 | 0 | 1 | 6 | 0.25 | 0.25 | 0.214286 | 43 |
| *lsd1-2* | 5 | 3 | 19 | 0 | 1 | 3 | 0.173913 | 0.173913043 | 0.130435 | 43 |
| *lsd1-2* | 3 | 1 | 15 | 0 | 0 | 1 | 0.0625 | 0.0625 | 0.0625 | 15 |
| *lsd1-2* | 4 | 1 | 13 | 3 | 1 | 2 | 0.315789 | 0.157894737 | 0.105263 | 15 |
| *lsd1-2* | 5 | 2 | 18 | 1 | 0 | 0 | 0.052632 | 0 | 0 | 15 |
| *lsd1-2* | 6 | 1 | 24 | 0 | 1 | 0 | 0.04 | 0.04 | 0 | 15 |
| *lsd1-2* | 7 | 4 | 22 | 0 | 2 | 2 | 0.153846 | 0.153846154 | 0.076923 | 15 |
| *lsd1-2* | 1 | 5 | 25 | 0 | 1 | 1 | 0.074074 | 0.074074074 | 0.037037 | 22 |
| *lsd1-2* | 3 | 5 | 25 | 1 | 1 | 2 | 0.137931 | 0.103448276 | 0.068966 | 22 |
| *lsd1-2* | 4 | 4 | 21 | 0 | 2 | 4 | 0.222222 | 0.222222222 | 0.148148 | 22 |
| *lsd1-2* | 6 | 4 | 24 | 0 | 1 | 2 | 0.111111 | 0.111111111 | 0.074074 | 22 |
| *lsd1-2* | 2 | 2 | 16 | 2 | 0 | 0 | 0.111111 | 0 | 0 | 23 |
| *lsd1-2* | 7 | 2 | 20 | 1 | 0 | 0 | 0.047619 | 0 | 0 | 23 |
| *lsd1-2* | 1 | 2 | 12 | 2 | 2 | 1 | 0.294118 | 0.176470588 | 0.058824 | 41 |
| *lsd1-2* | 2 | 4 | 23 | 0 | 2 | 1 | 0.115385 | 0.115384615 | 0.038462 | 41 |
| *lsd1-2* | 6 | 3 | 16 | 0 | 1 | 5 | 0.272727 | 0.272727273 | 0.227273 | 41 |
| *lsd1-2* | 7 | 2 | 16 | 1 | 2 | 1 | 0.2 | 0.15 | 0.05 | 41 |
| *lsd1-2* | 3 | 1 | 11 | 1 | 0 | 0 | 0.083333 | 0 | 0 | 42 |
| *lsd1-2* | 3 | 4 | 9 | 0 | 0 | 2 | 0.181818 | 0.181818182 | 0.181818 | 42 |
| *lsd1-2* | 4 | 3 | 12 | 0 | 2 | 4 | 0.333333 | 0.333333333 | 0.222222 | 42 |
| *lsd1-2* | 5 | 3 | 7 | 0 | 2 | 1 | 0.3 | 0.3 | 0.1 | 42 |
| *lsu2-379* | 1 | 4 | 28 | 1 | 5 | 0 | 0.176471 | 0.147058824 | 0 | 7 |
| *lsu2-379* | 2 | 3 | 24 | 0 | 0 | 1 | 0.04 | 0.04 | 0.04 | 7 |
| *lsu2-379* | 4 | 4 | 33 | 0 | 2 | 1 | 0.083333 | 0.083333333 | 0.027778 | 7 |
| *lsu2-379* | 5 | 5 | 29 | 0 | 4 | 1 | 0.147059 | 0.147058824 | 0.029412 | 7 |
| *lsu2-379* | 6 | 2 | 30 | 0 | 3 | 1 | 0.117647 | 0.117647059 | 0.029412 | 7 |
| *lsu2-379* | 1 | 2 | 14 | 0 | 0 | 4 | 0.222222 | 0.222222222 | 0.222222 | 16 |
| *lsu2-379* | 2 | 3 | 27 | 1 | 4 | 3 | 0.228571 | 0.2 | 0.085714 | 16 |
| *lsu2-379* | 3 | 1 | 26 | 3 | 2 | 4 | 0.257143 | 0.171428571 | 0.114286 | 16 |
| *lsu2-379* | 4 | 1 | 39 | 2 | 1 | 0 | 0.071429 | 0.023809524 | 0 | 16 |
| *lsu2-379* | 5 | 2 | 32 | 2 | 0 | 1 | 0.085714 | 0.028571429 | 0.028571 | 16 |
| *lsu2-379* | 5 | 5 | 27 | 0 | 3 | 0 | 0.1 | 0.1 | 0 | 16 |
| *lsu2-379* | 6 | 1 | 40 | 0 | 3 | 2 | 0.111111 | 0.111111111 | 0.044444 | 16 |
| *lsu2-379* | 1 | 1 | 25 | 2 | 1 | 2 | 0.166667 | 0.1 | 0.066667 | 17 |
| *lsu2-379* | 1 | 5 | 18 | 0 | 3 | 2 | 0.217391 | 0.217391304 | 0.086957 | 17 |
| *lsu2-379* | 2 | 2 | 26 | 0 | 2 | 1 | 0.103448 | 0.103448276 | 0.034483 | 17 |
| *lsu2-379* | 3 | 3 | 20 | 0 | 7 | 1 | 0.285714 | 0.285714286 | 0.035714 | 17 |
| *lsu2-379* | 4 | 1 | 26 | 1 | 5 | 1 | 0.212121 | 0.181818182 | 0.030303 | 17 |
| *lsu2-379* | 5 | 2 | 31 | 0 | 0 | 2 | 0.060606 | 0.060606061 | 0.060606 | 17 |
| *lsu2-379* | 6 | 4 | 31 | 0 | 6 | 4 | 0.243902 | 0.243902439 | 0.097561 | 17 |
| *lsu2-379* | 8 | 3 | 24 | 0 | 3 | 0 | 0.111111 | 0.111111111 | 0 | 17 |
| *lsu2-379* | 8 | 5 | 23 | 1 | 0 | 3 | 0.148148 | 0.111111111 | 0.111111 | 17 |
| *lsu2-392* |  |  | 15 | 1 | 2 | 1 | 0.210526 | 0.157894737 | 0.052632 | 4 |
| *lsu2-392* |  |  | 27 | 1 | 10 | 1 | 0.307692 | 0.282051282 | 0.025641 | 4 |
| *lsu2-392* |  |  | 26 | 1 | 8 | 3 | 0.315789 | 0.289473684 | 0.078947 | 4 |
| *lsu2-392* | 1 | 3 | 22 | 0 | 6 | 3 | 0.290323 | 0.290322581 | 0.096774 | 33 |
| *lsu2-392* | 2 | 4 | 18 | 1 | 0 | 1 | 0.1 | 0.05 | 0.05 | 33 |
| *lsu2-392* | 3 | 4 | 24 | 0 | 1 | 2 | 0.111111 | 0.111111111 | 0.074074 | 33 |
| *lsu2-392* | 4 | 3 | 9 | 0 | 3 | 1 | 0.307692 | 0.307692308 | 0.076923 | 33 |
| *lsu2-392* | 5 | 2 | 26 | 0 | 1 | 1 | 0.071429 | 0.071428571 | 0.035714 | 33 |
| *lsu2-392* | 6 | 3 | 21 | 0 | 5 | 4 | 0.3 | 0.3 | 0.133333 | 33 |
| *lsu2-392* | 7 | 2 | 25 | 2 | 0 | 3 | 0.166667 | 0.1 | 0.1 | 33 |
| *lsu2-392* | 3 | 1 | 23 | 0 | 0 | 3 | 0.115385 | 0.115384615 | 0.115385 | 34 |
| *lsu2-392* | 4 | 2 | 25 | 0 | 1 | 4 | 0.166667 | 0.166666667 | 0.133333 | 34 |
| *lsu2-392* | 5 | 5 | 17 | 0 | 0 | 3 | 0.15 | 0.15 | 0.15 | 34 |
| *lsu2-392* | 6 | 4 | 18 | 0 | 0 | 1 | 0.052632 | 0.052631579 | 0.052632 | 34 |
| *lsu2-392* | 1 | 3 | 21 | 2 | 4 | 3 | 0.3 | 0.233333333 | 0.1 | 15 |
| *lsu2-392* | 2 | 3 | 14 | 2 | 0 | 1 | 0.176471 | 0.058823529 | 0.058824 | 15 |
| *lsu2-392* | 3 | 4 | 22 | 1 | 1 | 2 | 0.153846 | 0.115384615 | 0.076923 | 15 |
| *lsu2-392* | 4 | 2 | 13 | 3 | 0 | 1 | 0.235294 | 0.058823529 | 0.058824 | 15 |
| *lsu2-392* | 5 | 3 | 21 | 3 | 1 | 2 | 0.222222 | 0.111111111 | 0.074074 | 15 |
| *lsu2-392* | 6 | 4 | 22 | 0 | 3 | 2 | 0.185185 | 0.185185185 | 0.074074 | 15 |
| *lsu2-392* | 7 | 3 | 21 | 1 | 2 | 3 | 0.222222 | 0.185185185 | 0.111111 | 15 |
| *lsu2-392* | 8 | 1 | 20 | 2 | 0 | 1 | 0.130435 | 0.043478261 | 0.043478 | 15 |
| *mkk1/2* | 1 | 2 | 23 | 0 | 1 | 3 | 0.148148 | 0.148148148 | 0.111111 | 10 |
| *mkk1/2* | 2 | 2 | 16 | 2 | 3 | 1 | 0.272727 | 0.181818182 | 0.045455 | 10 |
| *mkk1/2* | 3 | 5 | 30 | 1 | 0 | 0 | 0.032258 | 0 | 0 | 10 |
| *mkk1/2* | 4 | 2 | 20 | 1 | 4 | 0 | 0.2 | 0.16 | 0 | 10 |
| *mkk1/2* | 5 | 1 | 18 | 0 | 2 | 1 | 0.142857 | 0.142857143 | 0.047619 | 10 |
| *mkk1/2* | 6 | 1 | 14 | 0 | 0 | 1 | 0.066667 | 0.066666667 | 0.066667 | 10 |
| *mkk1/2* | 1 | 2 | 25 | 0 | 5 | 1 | 0.193548 | 0.193548387 | 0.032258 | 14 |
| *mkk1/2* | 1 | 3 | 17 | 1 | 1 | 4 | 0.26087 | 0.217391304 | 0.173913 | 14 |
| *mkk1/2* | 2 | 3 | 24 | 1 | 0 | 3 | 0.142857 | 0.107142857 | 0.107143 | 14 |
| *mkk1/2* | 3 | 2 | 27 | 0 | 3 | 3 | 0.181818 | 0.181818182 | 0.090909 | 14 |
| *mkk1/2* | 4 | 1 | 21 | 1 | 2 | 7 | 0.322581 | 0.290322581 | 0.225806 | 14 |
| *mkk1/2* | 6 | 5 | 23 | 0 | 1 | 1 | 0.08 | 0.08 | 0.04 | 14 |
| *mkk1/2* | 8 | 1 | 27 | 0 | 2 | 1 | 0.1 | 0.1 | 0.033333 | 14 |
| *mkk1/2* | 8 | 5 | 25 | 1 | 1 | 3 | 0.166667 | 0.133333333 | 0.1 | 14 |
| *mlo2-5/pen2-3* | 1 | 3 | 16 | 0 | 1 | 3 | 0.2 | 0.2 | 0.15 | 8 |
| *mlo2-5/pen2-3* | 2 | 2 | 18 | 1 | 2 | 0 | 0.142857 | 0.095238095 | 0 | 8 |
| *mlo2-5/pen2-3* | 3 | 1 | 12 | 1 | 2 | 1 | 0.25 | 0.1875 | 0.0625 | 8 |
| *mlo2-5/pen2-3* | 4 | 1 | 13 | 0 | 2 | 2 | 0.235294 | 0.235294118 | 0.117647 | 8 |
| *mlo2-5/pen2-3* | 5 | 3 | 14 | 0 | 4 | 4 | 0.363636 | 0.363636364 | 0.181818 | 8 |
| *mlo2-5/pen2-3* | 6 | 5 | 12 | 1 | 6 | 2 | 0.428571 | 0.380952381 | 0.095238 | 8 |
| *mlo2-5/pen2-3* | 7 | 2 | 12 | 0 | 2 | 1 | 0.2 | 0.2 | 0.066667 | 8 |
| *mlo2-5/pen2-3* | 1 | 4 | 31 | 0 | 3 | 1 | 0.114286 | 0.114285714 | 0.028571 | 28 |
| *mlo2-5/pen2-3* | 2 | 3 | 21 | 0 | 7 | 5 | 0.363636 | 0.363636364 | 0.151515 | 28 |
| *mlo2-5/pen2-3* | 3 | 3 | 19 | 0 | 3 | 0 | 0.136364 | 0.136363636 | 0 | 28 |
| *mlo2-5/pen2-3* | 4 | 2 | 14 | 0 | 3 | 2 | 0.263158 | 0.263157895 | 0.105263 | 28 |
| *mlo2-5/pen2-3* | 6 | 3 | 26 | 1 | 1 | 0 | 0.071429 | 0.035714286 | 0 | 28 |
| *mlo2-5/pen2-3* | 7 | 4 | 20 | 1 | 1 | 3 | 0.2 | 0.16 | 0.12 | 28 |
| *mlo2-5/pen2-3* | 5 | 2 | 13 | 2 | 2 | 0 | 0.235294 | 0.117647059 | 0 | 1 |
| *mlo2-5/pen2-3* | 3 | 4 | 18 | 0 | 0 | 1 | 0.052632 | 0.052631579 | 0.052632 | 1 |
| *mlo2-5/pen2-3* | 4 | 4 | 11 | 2 | 2 | 0 | 0.266667 | 0.133333333 | 0 | 1 |
| *mpk4* | 1 | 3 | 17 | 2 | 3 | 2 | 0.291667 | 0.208333333 | 0.083333 | 2 |
| *mpk4* | 2 | 1 | 16 | 4 | 2 | 0 | 0.272727 | 0.090909091 | 0 | 2 |
| *mpk4* | 3 | 5 | 26 | 0 | 1 | 2 | 0.103448 | 0.103448276 | 0.068966 | 2 |
| *mpk4* | 5 | 2 | 27 | 8 | 0 | 1 | 0.25 | 0.027777778 | 0.027778 | 2 |
| *mpk4* | 6 | 3 | 13 | 1 | 1 | 0 | 0.133333 | 0.066666667 | 0 | 2 |
| *mpk4* | 2 | 4 | 28 | 0 | 2 | 0 | 0.066667 | 0.066666667 | 0 | 21 |
| *mpk4* | 4 | 4 | 21 | 2 | 5 | 1 | 0.275862 | 0.206896552 | 0.034483 | 21 |
| *mpk4* | 5 | 2 | 8 | 0 | 1 | 1 | 0.2 | 0.2 | 0.1 | 21 |
| *mpk4* | 6 | 4 | 30 | 0 | 1 | 1 | 0.0625 | 0.0625 | 0.03125 | 21 |
| *mpk4* | 7 | 2 | 31 | 3 | 5 | 2 | 0.243902 | 0.170731707 | 0.04878 | 21 |
| *mpk4* | 1 | 2 | 37 | 0 | 1 | 0 | 0.026316 | 0.026315789 | 0 | 27 |
| *mpk4* | 2 | 5 | 11 | 0 | 0 | 0 | 0 | 0 | 0 | 27 |
| *mpk4* | 3 | 5 | 23 | 0 | 1 | 1 | 0.08 | 0.08 | 0.04 | 27 |
| *mpk4* | 4 | 1 | 10 | 1 | 3 | 0 | 0.285714 | 0.214285714 | 0 | 27 |
| *mpk4* | 5 | 3 | 32 | 0 | 0 | 2 | 0.058824 | 0.058823529 | 0.058824 | 27 |
| *mpk4* | 6 | 3 | 16 | 0 | 3 | 0 | 0.157895 | 0.157894737 | 0 | 27 |
| *NahG* | 1 | 1 | 15 | 0 | 0 | 4 | 0.210526 | 0.210526316 | 0.210526 | 13 |
| *NahG* | 2 | 2 | 25 | 0 | 1 | 5 | 0.193548 | 0.193548387 | 0.16129 | 13 |
| *NahG* | 2 | 5 | 25 | 0 | 1 | 0 | 0.038462 | 0.038461538 | 0 | 13 |
| *NahG* | 3 | 5 | 21 | 0 | 1 | 1 | 0.086957 | 0.086956522 | 0.043478 | 13 |
| *NahG* | 4 | 3 | 23 | 1 | 0 | 3 | 0.148148 | 0.111111111 | 0.111111 | 13 |
| *NahG* | 5 | 3 | 20 | 2 | 1 | 3 | 0.230769 | 0.153846154 | 0.115385 | 13 |
| *NahG* | 6 | 1 | 33 | 1 | 0 | 0 | 0.029412 | 0 | 0 | 13 |
| *NahG* | 6 | 4 | 33 | 0 | 3 | 5 | 0.195122 | 0.195121951 | 0.121951 | 13 |
| *NahG* | 1 | 4 | 29 | 0 | 2 | 1 | 0.09375 | 0.09375 | 0.03125 | 31 |
| *NahG* | 2 | 2 | 19 | 1 | 0 | 0 | 0.05 | 0 | 0 | 31 |
| *NahG* | 3 | 4 | 34 | 3 | 6 | 2 | 0.244444 | 0.177777778 | 0.044444 | 31 |
| *NahG* | 4 | 1 | 20 | 1 | 2 | 2 | 0.2 | 0.16 | 0.08 | 31 |
| *NahG* | 5 | 3 | 21 | 0 | 1 | 7 | 0.275862 | 0.275862069 | 0.241379 | 31 |
| *NahG* | 1 | 4 | 22 | 0 | 1 | 6 | 0.241379 | 0.24137931 | 0.206897 | 37 |
| *NahG* | 2 | 1 | 24 | 0 | 1 | 2 | 0.111111 | 0.111111111 | 0.074074 | 37 |
| *NahG* | 4 | 1 | 32 | 2 | 0 | 3 | 0.135135 | 0.081081081 | 0.081081 | 37 |
| *NahG* | 5 | 3 | 20 | 2 | 1 | 8 | 0.354839 | 0.290322581 | 0.258065 | 37 |
| *NahG* | 6 | 5 | 11 | 1 | 0 | 7 | 0.421053 | 0.368421053 | 0.368421 | 37 |
| *NahG* | 7 | 5 | 18 | 0 | 1 | 1 | 0.1 | 0.1 | 0.05 | 37 |
| *NahG* | 8 | 3 | 25 | 0 | 3 | 3 | 0.193548 | 0.193548387 | 0.096774 | 37 |
| *ndr1* | 1 | 5 | 24 | 0 | 0 | 1 | 0.04 | 0.04 | 0.04 | 37 |
| *ndr1* | 2 | 3 | 33 | 0 | 2 | 4 | 0.153846 | 0.153846154 | 0.102564 | 37 |
| *ndr1* | 3 | 3 | 33 | 1 | 4 | 7 | 0.266667 | 0.244444444 | 0.155556 | 37 |
| *ndr1* | 4 | 3 | 30 | 0 | 3 | 3 | 0.166667 | 0.166666667 | 0.083333 | 37 |
| *ndr1* | 6 | 4 | 22 | 0 | 0 | 4 | 0.153846 | 0.153846154 | 0.153846 | 37 |
| *ndr1* | 7 | 4 | 26 | 0 | 1 | 1 | 0.071429 | 0.071428571 | 0.035714 | 37 |
| *ndr1* | 8 | 5 | 34 | 1 | 2 | 1 | 0.105263 | 0.078947368 | 0.026316 | 37 |
| *ndr1* | 1 | 5 | 11 | 2 | 0 | 2 | 0.266667 | 0.133333333 | 0.133333 | 43 |
| *ndr1* | 2 | 3 | 12 | 0 | 1 | 1 | 0.142857 | 0.142857143 | 0.071429 | 43 |
| *ndr1* | 3 | 2 | 20 | 3 | 1 | 1 | 0.2 | 0.08 | 0.04 | 43 |
| *ndr1* | 4 | 5 | 20 | 0 | 0 | 2 | 0.090909 | 0.090909091 | 0.090909 | 43 |
| *ndr1* | 5 | 5 | 8 | 0 | 0 | 2 | 0.2 | 0.2 | 0.2 | 43 |
| *ndr1* | 6 | 3 | 21 | 0 | 2 | 1 | 0.125 | 0.125 | 0.041667 | 43 |
| *ndr1* | 8 | 2 | 45 | 1 | 1 | 2 | 0.081633 | 0.06122449 | 0.040816 | 43 |
| *ndr1* | 1 | 1 | 19 | 0 | 0 | 1 | 0.05 | 0.05 | 0.05 | 44 |
| *pad4* | 1 | 1 | 14 | 0 | 2 | 11 | 0.481481 | 0.481481481 | 0.407407 | 37 |
| *pad4* | 2 | 2 | 34 | 1 | 2 | 4 | 0.170732 | 0.146341463 | 0.097561 | 37 |
| *pad4* | 3 | 1 | 25 | 1 | 0 | 2 | 0.107143 | 0.071428571 | 0.071429 | 37 |
| *pad4* | 4 | 5 | 29 | 1 | 0 | 5 | 0.171429 | 0.142857143 | 0.142857 | 37 |
| *pad4* | 5 | 2 | 32 | 0 | 2 | 4 | 0.157895 | 0.157894737 | 0.105263 | 37 |
| *pad4* | 6 | 2 | 26 | 1 | 2 | 5 | 0.235294 | 0.205882353 | 0.147059 | 37 |
| *pad4* | 7 | 1 | 17 | 1 | 0 | 0 | 0.055556 | 0 | 0 | 37 |
| *pad4* | 8 | 1 | 18 | 0 | 1 | 0 | 0.052632 | 0.052631579 | 0 | 37 |
| *pad4* | 2 | 5 | 17 | 0 | 1 | 1 | 0.105263 | 0.105263158 | 0.052632 | 43 |
| *pad4* | 3 | 5 | 30 | 0 | 1 | 1 | 0.0625 | 0.0625 | 0.03125 | 43 |
| *pad4* | 5 | 4 | 14 | 0 | 1 | 5 | 0.3 | 0.3 | 0.25 | 43 |
| *pad4* | 6 | 1 | 15 | 0 | 1 | 3 | 0.210526 | 0.210526316 | 0.157895 | 43 |
| *pad4* | 7 | 2 | 24 | 0 | 0 | 4 | 0.142857 | 0.142857143 | 0.142857 | 43 |
| *pad4* | 1 | 3 | 23 | 1 | 1 | 2 | 0.148148 | 0.111111111 | 0.074074 | 44 |
| *pad4* | 7 | 2 | 39 | 0 | 2 | 2 | 0.093023 | 0.093023256 | 0.046512 | 44 |
| *pad4/ndr1* | 1 | 3 | 19 | 0 | 1 | 1 | 0.095238 | 0.095238095 | 0.047619 | 18 |
| *pad4/ndr1* | 2 | 4 | 21 | 2 | 2 | 4 | 0.275862 | 0.206896552 | 0.137931 | 18 |
| *pad4/ndr1* | 3 | 3 | 28 | 2 | 1 | 1 | 0.125 | 0.0625 | 0.03125 | 18 |
| *pad4/ndr1* | 4 | 5 | 34 | 1 | 2 | 5 | 0.190476 | 0.166666667 | 0.119048 | 18 |
| *pad4/ndr1* | 5 | 3 | 34 | 2 | 3 | 2 | 0.170732 | 0.12195122 | 0.04878 | 18 |
| *pad4/ndr1* | 6 | 5 | 26 | 1 | 1 | 6 | 0.235294 | 0.205882353 | 0.176471 | 18 |
| *pad4/ndr1* | 7 | 1 | 16 | 0 | 0 | 1 | 0.058824 | 0.058823529 | 0.058824 | 18 |
| *pad4/ndr1* | 8 | 1 | 29 | 0 | 0 | 1 | 0.033333 | 0.033333333 | 0.033333 | 18 |
| *pad4/ndr1* | 1 | 4 | 34 | 3 | 1 | 9 | 0.276596 | 0.212765957 | 0.191489 | 21 |
| *pad4/ndr1* | 2 | 5 | 22 | 3 | 3 | 2 | 0.266667 | 0.166666667 | 0.066667 | 21 |
| *pad4/ndr1* | 3 | 2 | 27 | 2 | 0 | 0 | 0.068966 | 0 | 0 | 21 |
| *pad4/ndr1* | 4 | 3 | 31 | 1 | 5 | 3 | 0.225 | 0.2 | 0.075 | 21 |
| *pad4/ndr1* | 5 | 1 | 25 | 1 | 6 | 7 | 0.358974 | 0.333333333 | 0.179487 | 21 |
| *pad4/ndr1* | 6 | 2 | 44 | 1 | 4 | 4 | 0.169811 | 0.150943396 | 0.075472 | 21 |
| *pad4/ndr1* | 7 | 5 | 31 | 1 | 6 | 5 | 0.27907 | 0.255813953 | 0.116279 | 21 |
| *pen2-3* | 1 | 4 | 22 | 1 | 1 | 0 | 0.083333 | 0.041666667 | 0 | 18 |
| *pen2-3* | 2 | 2 | 34 | 1 | 0 | 0 | 0.028571 | 0 | 0 | 18 |
| *pen2-3* | 3 | 5 | 23 | 0 | 1 | 5 | 0.206897 | 0.206896552 | 0.172414 | 18 |
| *pen2-3* | 4 | 3 | 13 | 0 | 7 | 6 | 0.5 | 0.5 | 0.230769 | 18 |
| *pen2-3* | 5 | 5 | 14 | 1 | 0 | 4 | 0.263158 | 0.210526316 | 0.210526 | 18 |
| *pen2-3* | 6 | 2 | 22 | 0 | 4 | 3 | 0.241379 | 0.24137931 | 0.103448 | 18 |
| *pen2-3* | 7 | 3 | 43 | 0 | 8 | 8 | 0.271186 | 0.271186441 | 0.135593 | 18 |
| *pen2-3* | 8 | 2 | 39 | 0 | 3 | 6 | 0.1875 | 0.1875 | 0.125 | 18 |
| *pen2-3* | 1 | 3 | 19 | 1 | 3 | 7 | 0.366667 | 0.333333333 | 0.233333 | 25 |
| *pen2-3* | 2 | 4 | 31 | 1 | 0 | 3 | 0.114286 | 0.085714286 | 0.085714 | 25 |
| *pen2-3* | 3 | 5 | 20 | 1 | 5 | 2 | 0.285714 | 0.25 | 0.071429 | 25 |
| *pen2-3* | 4 | 2 | 24 | 1 | 1 | 3 | 0.172414 | 0.137931034 | 0.103448 | 25 |
| *pen2-3* | 5 | 2 | 32 | 1 | 2 | 9 | 0.272727 | 0.25 | 0.204545 | 25 |
| *pen2-3* | 6 | 3 | 25 | 0 | 0 | 2 | 0.074074 | 0.074074074 | 0.074074 | 25 |
| *pen2-3* | 6 | 4 | 28 | 0 | 2 | 6 | 0.222222 | 0.222222222 | 0.166667 | 25 |
| *pen2-3* | 3 | 2 | 20 | 1 | 6 | 0 | 0.259259 | 0.222222222 | 0 | 28 |
| *pen2-3* | 4 | 5 | 20 | 0 | 1 | 0 | 0.047619 | 0.047619048 | 0 | 28 |
| *pen2-3* | 7 | 3 | 14 | 11 | 2 | 1 | 0.5 | 0.107142857 | 0.035714 | 28 |
| *pepr1/2* | 1 | 4 | 18 | 0 | 0 | 2 | 0.1 | 0.1 | 0.1 | 8 |
| *pepr1/2* | 2 | 3 | 16 | 0 | 5 | 0 | 0.238095 | 0.238095238 | 0 | 8 |
| *pepr1/2* | 3 | 4 | 16 | 1 | 3 | 5 | 0.36 | 0.32 | 0.2 | 8 |
| *pepr1/2* | 5 | 2 | 12 | 1 | 2 | 0 | 0.2 | 0.133333333 | 0 | 8 |
| *pepr1/2* | 6 | 1 | 19 | 0 | 7 | 1 | 0.296296 | 0.296296296 | 0.037037 | 8 |
| *pepr1/2* | 7 | 3 | 16 | 1 | 1 | 1 | 0.157895 | 0.105263158 | 0.052632 | 8 |
| *pepr1/2* | 1 | 1 | 14 | 0 | 4 | 0 | 0.222222 | 0.222222222 | 0 | 19 |
| *pepr1/2* | 2 | 4 | 18 | 0 | 1 | 5 | 0.25 | 0.25 | 0.208333 | 19 |
| *pepr1/2* | 3 | 1 | 20 | 0 | 3 | 1 | 0.166667 | 0.166666667 | 0.041667 | 19 |
| *pepr1/2* | 5 | 3 | 20 | 1 | 3 | 2 | 0.230769 | 0.192307692 | 0.076923 | 19 |
| *pepr1/2* | 6 | 2 | 16 | 0 | 0 | 3 | 0.157895 | 0.157894737 | 0.157895 | 19 |
| *pepr1/2* | 7 | 1 | 14 | 0 | 0 | 2 | 0.125 | 0.125 | 0.125 | 19 |
| *pepr1/2* | 8 | 3 | 16 | 0 | 0 | 2 | 0.111111 | 0.111111111 | 0.111111 | 19 |
| *pepr1/2* | 1 | 3 | 36 | 1 | 7 | 2 | 0.217391 | 0.195652174 | 0.043478 | 28 |
| *pepr1/2* | 2 | 2 | 25 | 1 | 3 | 1 | 0.166667 | 0.133333333 | 0.033333 | 28 |
| *pepr1/2* | 4 | 5 | 30 | 0 | 10 | 10 | 0.4 | 0.4 | 0.2 | 28 |
| *pepr1/2* | 5 | 2 | 31 | 0 | 2 | 0 | 0.060606 | 0.060606061 | 0 | 28 |
| *pepr1/2* | 6 | 4 | 30 | 0 | 2 | 3 | 0.142857 | 0.142857143 | 0.085714 | 28 |
| *pepr1/2* | 7 | 2 | 21 | 1 | 1 | 0 | 0.086957 | 0.043478261 | 0 | 28 |
| *pepr1/2* | 1 | 1 | 24 | 1 | 2 | 4 | 0.225806 | 0.193548387 | 0.129032 | 32 |
| *pepr1/2* | 2 | 3 | 30 | 0 | 2 | 3 | 0.142857 | 0.142857143 | 0.085714 | 32 |
| *pepr1/2* | 3 | 3 | 7 | 0 | 1 | 1 | 0.222222 | 0.222222222 | 0.111111 | 32 |
| *pepr1/2* | 4 | 5 | 31 | 1 | 4 | 3 | 0.205128 | 0.179487179 | 0.076923 | 32 |
| *pepr1/2* | 5 | 4 | 40 | 0 | 2 | 3 | 0.111111 | 0.111111111 | 0.066667 | 32 |
| *pepr1/2* | 6 | 1 | 25 | 0 | 2 | 3 | 0.166667 | 0.166666667 | 0.1 | 32 |
| *pfd6* | 1 | 2 | 13 | 2 | 0 | 1 | 0.1875 | 0.0625 | 0.0625 | 9 |
| *pfd6* | 2 | 4 | 18 | 0 | 4 | 1 | 0.217391 | 0.217391304 | 0.043478 | 9 |
| *pfd6* | 3 | 3 | 14 | 6 | 3 | 3 | 0.461538 | 0.230769231 | 0.115385 | 9 |
| *pfd6* | 6 | 5 | 29 | 5 | 9 | 0 | 0.325581 | 0.209302326 | 0 | 9 |
| *pfd6* | 1 | 3 | 23 | 1 | 0 | 1 | 0.08 | 0.04 | 0.04 | 10 |
| *pfd6* | 2 | 1 | 19 | 0 | 1 | 1 | 0.095238 | 0.095238095 | 0.047619 | 10 |
| *pfd6* | 5 | 4 | 22 | 0 | 3 | 1 | 0.153846 | 0.153846154 | 0.038462 | 10 |
| *pfd6* | 6 | 5 | 13 | 0 | 1 | 1 | 0.133333 | 0.133333333 | 0.066667 | 10 |
| *pfd6* | 2 | 3 | 17 | 0 | 1 | 1 | 0.105263 | 0.105263158 | 0.052632 | 21 |
| *pfd6* | 3 | 4 | 25 | 3 | 4 | 2 | 0.264706 | 0.176470588 | 0.058824 | 21 |
| *pfd6* | 4 | 5 | 18 | 0 | 8 | 2 | 0.357143 | 0.357142857 | 0.071429 | 21 |
| *pfd6* | 5 | 3 | 12 | 2 | 2 | 2 | 0.333333 | 0.222222222 | 0.111111 | 21 |
| *pfd6* | 6 | 5 | 39 | 8 | 10 | 3 | 0.35 | 0.216666667 | 0.05 | 21 |
| *pfd6* | 7 | 4 | 15 | 1 | 8 | 2 | 0.423077 | 0.384615385 | 0.076923 | 21 |
| *pfd6* | 1 | 3 | 22 | 0 | 2 | 1 | 0.12 | 0.12 | 0.04 | 27 |
| *pfd6* | 2 | 2 | 29 | 4 | 3 | 3 | 0.25641 | 0.153846154 | 0.076923 | 27 |
| *pfd6* | 5 | 4 | 18 | 1 | 1 | 3 | 0.217391 | 0.173913043 | 0.130435 | 27 |
| *pfd6* | 7 | 5 | 18 | 0 | 0 | 0 | 0 | 0 | 0 | 27 |
| *dde2/pad4/sid2/ein2* | 1 | 1 | 22 | 0 | 3 | 2 | 0.185185 | 0.185185185 | 0.074074 | 40 |
| *dde2/pad4/sid2/ein2* | 3 | 1 | 13 | 0 | 2 | 2 | 0.235294 | 0.235294118 | 0.117647 | 40 |
| *dde2/pad4/sid2/ein2* | 4 | 2 | 17 | 0 | 4 | 4 | 0.32 | 0.32 | 0.16 | 40 |
| *dde2/pad4/sid2/ein2* | 5 | 5 | 8 | 1 | 0 | 0 | 0.111111 | 0 | 0 | 40 |
| *dde2/pad4/sid2/ein2* | 1 | 4 | 20 | 0 | 0 | 2 | 0.090909 | 0.090909091 | 0.090909 | 3 |
| *dde2/pad4/sid2/ein2* | 2 | 1 | 23 | 0 | 4 | 2 | 0.206897 | 0.206896552 | 0.068966 | 3 |
| *dde2/pad4/sid2/ein2* | 3 | 5 | 23 | 0 | 1 | 1 | 0.08 | 0.08 | 0.04 | 3 |
| *dde2/pad4/sid2/ein2* | 4 | 4 | 10 | 1 | 3 | 2 | 0.375 | 0.3125 | 0.125 | 3 |
| *dde2/pad4/sid2/ein2* | 5 | 2 | 16 | 2 | 5 | 3 | 0.384615 | 0.307692308 | 0.115385 | 3 |
| *dde2/pad4/sid2/ein2* | 6 | 1 | 19 | 1 | 1 | 1 | 0.136364 | 0.090909091 | 0.045455 | 3 |
| *rar1* |  |  | 16 | 0 | 1 | 0 | 0.058824 | 0.058823529 | 0 | 4 |
| *rar1* |  |  | 10 | 1 | 6 | 2 | 0.473684 | 0.421052632 | 0.105263 | 4 |
| *rar1* |  |  | 23 | 0 | 2 | 1 | 0.115385 | 0.115384615 | 0.038462 | 4 |
| *rar1* |  |  | 14 | 2 | 7 | 2 | 0.44 | 0.36 | 0.08 | 4 |
| *rar1* |  |  | 29 | 0 | 5 | 3 | 0.216216 | 0.216216216 | 0.081081 | 4 |
| *rar1* | 1 | 5 | 25 | 3 | 2 | 1 | 0.193548 | 0.096774194 | 0.032258 | 16 |
| *rar1* | 2 | 2 | 33 | 0 | 2 | 2 | 0.108108 | 0.108108108 | 0.054054 | 16 |
| *rar1* | 3 | 2 | 35 | 3 | 5 | 3 | 0.23913 | 0.173913043 | 0.065217 | 16 |
| *rar1* | 3 | 5 | 34 | 3 | 4 | 6 | 0.276596 | 0.212765957 | 0.12766 | 16 |
| *rar1* | 4 | 2 | 38 | 3 | 4 | 1 | 0.173913 | 0.108695652 | 0.021739 | 16 |
| *rar1* | 5 | 4 | 35 | 0 | 1 | 2 | 0.078947 | 0.078947368 | 0.052632 | 16 |
| *rar1* | 6 | 4 | 23 | 0 | 0 | 1 | 0.041667 | 0.041666667 | 0.041667 | 16 |
| *rar1* | 1 | 4 | 24 | 0 | 0 | 1 | 0.04 | 0.04 | 0.04 | 17 |
| *rar1* | 2 | 1 | 25 | 0 | 1 | 0 | 0.038462 | 0.038461538 | 0 | 17 |
| *rar1* | 3 | 2 | 20 | 0 | 3 | 3 | 0.230769 | 0.230769231 | 0.115385 | 17 |
| *rar1* | 3 | 5 | 23 | 1 | 4 | 0 | 0.178571 | 0.142857143 | 0 | 17 |
| *rar1* | 4 | 3 | 25 | 1 | 0 | 1 | 0.074074 | 0.037037037 | 0.037037 | 17 |
| *rar1* | 4 | 5 | 17 | 4 | 2 | 0 | 0.26087 | 0.086956522 | 0 | 17 |
| *rar1* | 6 | 3 | 32 | 0 | 4 | 3 | 0.179487 | 0.179487179 | 0.076923 | 17 |
| *rar1* | 8 | 4 | 24 | 1 | 1 | 1 | 0.111111 | 0.074074074 | 0.037037 | 17 |
| *rdr triple* | 1 | 2 | 23 | 2 | 5 | 3 | 0.30303 | 0.242424242 | 0.090909 | 15 |
| *rdr triple* | 2 | 2 | 31 | 0 | 4 | 7 | 0.261905 | 0.261904762 | 0.166667 | 15 |
| *rdr triple* | 3 | 3 | 13 | 1 | 3 | 2 | 0.315789 | 0.263157895 | 0.105263 | 15 |
| *rdr triple* | 4 | 3 | 27 | 0 | 0 | 3 | 0.1 | 0.1 | 0.1 | 15 |
| *rdr triple* | 5 | 4 | 21 | 1 | 1 | 4 | 0.222222 | 0.185185185 | 0.148148 | 15 |
| *rdr triple* | 6 | 5 | 28 | 4 | 7 | 1 | 0.3 | 0.2 | 0.025 | 15 |
| *rdr triple* | 7 | 5 | 17 | 0 | 1 | 3 | 0.190476 | 0.19047619 | 0.142857 | 15 |
| *rdr triple* | 8 | 5 | 21 | 0 | 4 | 3 | 0.25 | 0.25 | 0.107143 | 15 |
| *rdr triple* | 1 | 5 | 26 | 1 | 5 | 1 | 0.212121 | 0.181818182 | 0.030303 | 19 |
| *rdr triple* | 4 | 5 | 5 | 0 | 0 | 2 | 0.285714 | 0.285714286 | 0.285714 | 19 |
| *rdr triple* | 5 | 2 | 7 | 0 | 1 | 0 | 0.125 | 0.125 | 0 | 19 |
| *rdr triple* | 6 | 4 | 19 | 1 | 1 | 1 | 0.136364 | 0.090909091 | 0.045455 | 19 |
| *rdr triple* | 7 | 5 | 16 | 1 | 2 | 6 | 0.36 | 0.32 | 0.24 | 19 |
| *rdr triple* | 8 | 1 | 10 | 0 | 0 | 2 | 0.166667 | 0.166666667 | 0.166667 | 19 |
| *rdr triple* | 1 | 5 | 20 | 0 | 4 | 1 | 0.2 | 0.2 | 0.04 | 1 |
| *rdr triple* | 3 | 2 | 24 | 0 | 1 | 5 | 0.2 | 0.2 | 0.166667 | 1 |
| *rdr triple* | 4 | 1 | 13 | 1 | 0 | 4 | 0.277778 | 0.222222222 | 0.222222 | 1 |
| *rdr6-11* | 1 | 1 | 19 | 0 | 1 | 2 | 0.136364 | 0.136363636 | 0.090909 | 35 |
| *rdr6-11* | 2 | 2 | 30 | 0 | 0 | 0 | 0 | 0 | 0 | 35 |
| *rdr6-11* | 3 | 1 | 13 | 0 | 3 | 2 | 0.277778 | 0.277777778 | 0.111111 | 35 |
| *rdr6-11* | 4 | 3 | 25 | 1 | 1 | 1 | 0.107143 | 0.071428571 | 0.035714 | 35 |
| *rdr6-11* | 5 | 3 | 29 | 0 | 2 | 0 | 0.064516 | 0.064516129 | 0 | 35 |
| *rdr6-11* | 6 | 1 | 15 | 0 | 0 | 2 | 0.117647 | 0.117647059 | 0.117647 | 35 |
| *rdr6-11* | 7 | 4 | 17 | 0 | 3 | 0 | 0.15 | 0.15 | 0 | 35 |
| *rdr6-11* | 1 | 1 | 19 | 0 | 1 | 2 | 0.136364 | 0.136363636 | 0.090909 | 36 |
| *rdr6-11* | 2 | 2 | 30 | 0 | 0 | 0 | 0 | 0 | 0 | 36 |
| *rdr6-11* | 3 | 1 | 13 | 0 | 3 | 2 | 0.277778 | 0.277777778 | 0.111111 | 36 |
| *rdr6-11* | 4 | 3 | 25 | 1 | 1 | 1 | 0.107143 | 0.071428571 | 0.035714 | 36 |
| *rdr6-11* | 5 | 3 | 29 | 0 | 2 | 0 | 0.064516 | 0.064516129 | 0 | 36 |
| *rdr6-11* | 6 | 1 | 15 | 0 | 0 | 2 | 0.117647 | 0.117647059 | 0.117647 | 36 |
| *rdr6-11* | 7 | 4 | 17 | 0 | 3 | 0 | 0.15 | 0.15 | 0 | 36 |
| *rpm1* | 1 | 5 | 21 | 1 | 3 | 2 | 0.222222 | 0.185185185 | 0.074074 | 21 |
| *rpm1* | 2 | 2 | 22 | 2 | 8 | 2 | 0.352941 | 0.294117647 | 0.058824 | 21 |
| *rpm1* | 4 | 2 | 23 | 0 | 4 | 4 | 0.258065 | 0.258064516 | 0.129032 | 21 |
| *rpm1* | 5 | 5 | 32 | 3 | 4 | 0 | 0.179487 | 0.102564103 | 0 | 21 |
| *rpm1* | 6 | 3 | 31 | 2 | 6 | 3 | 0.261905 | 0.214285714 | 0.071429 | 21 |
| *rpm1* | 7 | 1 | 25 | 0 | 1 | 1 | 0.074074 | 0.074074074 | 0.037037 | 21 |
| *rpm1* | 1 | 5 | 19 | 0 | 5 | 6 | 0.366667 | 0.366666667 | 0.2 | 23 |
| *rpm1* | 2 | 1 | 4 | 1 | 0 | 0 | 0.2 | 0 | 0 | 23 |
| *rpm1* | 3 | 5 | 17 | 0 | 0 | 3 | 0.15 | 0.15 | 0.15 | 23 |
| *rpm1* | 4 | 4 | 31 | 1 | 5 | 0 | 0.162162 | 0.135135135 | 0 | 23 |
| *rpm1* | 5 | 2 | 21 | 1 | 0 | 6 | 0.25 | 0.214285714 | 0.214286 | 23 |
| *rpm1* | 6 | 5 | 15 | 0 | 4 | 4 | 0.347826 | 0.347826087 | 0.173913 | 23 |
| *rpm1* | 7 | 4 | 28 | 0 | 1 | 0 | 0.034483 | 0.034482759 | 0 | 23 |
| *rpm1* | 1 | 4 | 24 | 1 | 1 | 3 | 0.172414 | 0.137931034 | 0.103448 | 33 |
| *rpm1* | 2 | 2 | 17 | 2 | 5 | 5 | 0.413793 | 0.344827586 | 0.172414 | 33 |
| *rpm1* | 3 | 2 | 27 | 0 | 2 | 1 | 0.1 | 0.1 | 0.033333 | 33 |
| *rpm1* | 4 | 4 | 15 | 1 | 5 | 5 | 0.423077 | 0.384615385 | 0.192308 | 33 |
| *rpm1* | 5 | 1 | 25 | 0 | 3 | 0 | 0.107143 | 0.107142857 | 0 | 33 |
| *rpm1* | 6 | 4 | 20 | 0 | 7 | 0 | 0.259259 | 0.259259259 | 0 | 33 |
| *rpm1* | 7 | 5 | 18 | 0 | 1 | 4 | 0.217391 | 0.217391304 | 0.173913 | 33 |
| *sgt1* | 1 | 3 | 30 | 0 | 4 | 1 | 0.142857 | 0.142857143 | 0.028571 | 11 |
| *sgt1* | 2 | 2 | 34 | 0 | 1 | 2 | 0.081081 | 0.081081081 | 0.054054 | 11 |
| *sgt1* | 4 | 4 | 12 | 1 | 0 | 2 | 0.2 | 0.133333333 | 0.133333 | 11 |
| *sgt1* | 6 | 5 | 25 | 0 | 1 | 0 | 0.038462 | 0.038461538 | 0 | 11 |
| *sgt1* | 1 | 1 | 19 | 0 | 3 | 2 | 0.208333 | 0.208333333 | 0.083333 | 20 |
| *sgt1* | 2 | 4 | 33 | 0 | 3 | 5 | 0.195122 | 0.195121951 | 0.121951 | 20 |
| *sgt1* | 3 | 4 | 29 | 1 | 3 | 1 | 0.147059 | 0.117647059 | 0.029412 | 20 |
| *sgt1* | 4 | 2 | 11 | 1 | 4 | 2 | 0.388889 | 0.333333333 | 0.111111 | 20 |
| *sgt1* | 5 | 4 | 18 | 0 | 2 | 1 | 0.142857 | 0.142857143 | 0.047619 | 20 |
| *sgt1* | 6 | 3 | 31 | 2 | 7 | 11 | 0.392157 | 0.352941176 | 0.215686 | 20 |
| *sgt1* | 7 | 4 | 13 | 0 | 0 | 1 | 0.071429 | 0.071428571 | 0.071429 | 20 |
| *sgt1* | 8 | 3 | 35 | 1 | 5 | 2 | 0.186047 | 0.162790698 | 0.046512 | 20 |
| *sgt1* | 1 | 2 | 33 | 0 | 1 | 0 | 0.029412 | 0.029411765 | 0 | 31 |
| *sgt1* | 2 | 5 | 16 | 0 | 4 | 4 | 0.333333 | 0.333333333 | 0.166667 | 31 |
| *sgt1* | 3 | 2 | 34 | 0 | 7 | 4 | 0.244444 | 0.244444444 | 0.088889 | 31 |
| *sgt1* | 5 | 4 | 23 | 0 | 1 | 3 | 0.148148 | 0.148148148 | 0.111111 | 31 |
| *sgt1* | 6 | 2 | 34 | 1 | 3 | 1 | 0.128205 | 0.102564103 | 0.025641 | 31 |
| *sid2* | 1 | 3 | 7 | 0 | 1 | 1 | 0.222222 | 0.222222222 | 0.111111 | 12 |
| *sid2* | 2 | 5 | 11 | 0 | 0 | 3 | 0.214286 | 0.214285714 | 0.214286 | 12 |
| *sid2* | 3 | 2 | 23 | 0 | 0 | 2 | 0.08 | 0.08 | 0.08 | 12 |
| *sid2* | 4 | 2 | 18 | 0 | 1 | 1 | 0.1 | 0.1 | 0.05 | 12 |
| *sid2* | 5 | 2 | 14 | 0 | 0 | 1 | 0.066667 | 0.066666667 | 0.066667 | 12 |
| *sid2* | 7 | 3 | 20 | 0 | 0 | 2 | 0.090909 | 0.090909091 | 0.090909 | 12 |
| *sid2* | 7 | 5 | 27 | 0 | 0 | 4 | 0.129032 | 0.129032258 | 0.129032 | 12 |
| *sid2* | 1 | 3 | 33 | 1 | 1 | 1 | 0.083333 | 0.055555556 | 0.027778 | 30 |
| *sid2* | 2 | 3 | 25 | 0 | 0 | 0 | 0 | 0 | 0 | 30 |
| *sid2* | 3 | 2 | 21 | 0 | 3 | 4 | 0.25 | 0.25 | 0.142857 | 30 |
| *sid2* | 4 | 3 | 28 | 0 | 0 | 5 | 0.151515 | 0.151515152 | 0.151515 | 30 |
| *sid2* | 5 | 1 | 23 | 0 | 0 | 1 | 0.041667 | 0.041666667 | 0.041667 | 30 |
| *sid2* | 6 | 3 | 26 | 0 | 6 | 4 | 0.277778 | 0.277777778 | 0.111111 | 30 |
| *sid2* | 7 | 2 | 26 | 0 | 1 | 5 | 0.1875 | 0.1875 | 0.15625 | 30 |
| *sid2* | 8 | 2 | 21 | 1 | 9 | 2 | 0.363636 | 0.333333333 | 0.060606 | 30 |
| *tir1* | 1 | 5 | 12 | 2 | 3 | 1 | 0.333333 | 0.222222222 | 0.055556 | 7 |
| *tir1* | 2 | 4 | 27 | 2 | 5 | 0 | 0.205882 | 0.147058824 | 0 | 7 |
| *tir1* | 3 | 4 | 13 | 0 | 6 | 1 | 0.35 | 0.35 | 0.05 | 7 |
| *tir1* | 4 | 5 | 18 | 0 | 4 | 0 | 0.181818 | 0.181818182 | 0 | 7 |
| *tir1* | 5 | 1 | 23 | 1 | 1 | 0 | 0.08 | 0.04 | 0 | 7 |
| *tir1* | 6 | 4 | 34 | 1 | 5 | 1 | 0.170732 | 0.146341463 | 0.02439 | 7 |
| *tir1* | 1 | 2 | 22 | 1 | 1 | 2 | 0.153846 | 0.115384615 | 0.076923 | 22 |
| *tir1* | 3 | 3 | 32 | 0 | 3 | 3 | 0.157895 | 0.157894737 | 0.078947 | 22 |
| *tir1* | 4 | 5 | 42 | 0 | 0 | 4 | 0.086957 | 0.086956522 | 0.086957 | 22 |
| *tir1* | 5 | 3 | 25 | 1 | 1 | 3 | 0.166667 | 0.133333333 | 0.1 | 22 |
| *tir1* | 6 | 2 | 38 | 0 | 3 | 3 | 0.136364 | 0.136363636 | 0.068182 | 22 |
| *tir1* | 1 | 5 | 17 | 1 | 2 | 4 | 0.291667 | 0.25 | 0.166667 | 2 |
| *tir1* | 2 | 5 | 20 | 0 | 7 | 3 | 0.333333 | 0.333333333 | 0.1 | 2 |
| *tir1* | 3 | 3 | 19 | 0 | 1 | 4 | 0.208333 | 0.208333333 | 0.166667 | 2 |
| *tir1* | 4 | 2 | 16 | 1 | 2 | 2 | 0.238095 | 0.19047619 | 0.095238 | 2 |
| ***Plant*** | **Bag** | **Position** | **Unattached** | **S3** | **S4.1** | **S4.2** | **S3 rate** | **S4.1 rate** | **S4.2 rate** | **Experiment** |
| *arr triple* | 1 | 2 | 20 | 1 | 1 | 5 | 0.259259 | 0.222222222 | 0.185185 | 36 |
| *arr triple* | 2 | 3 | 29 | 1 | 1 | 2 | 0.121212 | 0.090909091 | 0.060606 | 36 |
| *arr triple* | 3 | 2 | 31 | 1 | 2 | 8 | 0.261905 | 0.238095238 | 0.190476 | 36 |
| *arr triple* | 5 | 1 | 26 | 0 | 5 | 4 | 0.257143 | 0.257142857 | 0.114286 | 36 |
| *arr triple* | 6 | 4 | 25 | 0 | 3 | 9 | 0.324324 | 0.324324324 | 0.243243 | 36 |
| *arr triple* | 7 | 5 | 17 | 0 | 2 | 5 | 0.291667 | 0.291666667 | 0.208333 | 36 |
| *arr triple* | 2 | 2 | 12 | 0 | 0 | 3 | 0.2 | 0.2 | 0.2 | 38 |
| *arr triple* | 4 | 4 | 6 | 1 | 0 | 2 | 0.333333 | 0.222222222 | 0.222222 | 38 |
| *arr triple* | 6 | 2 | 10 | 0 | 1 | 2 | 0.230769 | 0.230769231 | 0.153846 | 38 |
| *arr triple* | 2 | 4 | 22 | 0 | 3 | 7 | 0.3125 | 0.3125 | 0.21875 | 39 |
| *arr triple* | 3 | 3 | 24 | 3 | 3 | 1 | 0.225806 | 0.129032258 | 0.032258 | 39 |
| *arr triple* | 4 | 4 | 22 | 2 | 3 | 6 | 0.333333 | 0.272727273 | 0.181818 | 39 |
| *arr triple* | 5 | 4 | 8 | 0 | 7 | 8 | 0.652174 | 0.652173913 | 0.347826 | 39 |
| *arr triple* | 6 | 3 | 12 | 0 | 1 | 11 | 0.5 | 0.5 | 0.458333 | 39 |
| *arr triple* | 7 | 3 | 28 | 1 | 5 | 8 | 0.333333 | 0.30952381 | 0.190476 | 39 |
| *arr triple* | 8 | 5 | 16 | 0 | 2 | 3 | 0.238095 | 0.238095238 | 0.142857 | 39 |
| *eds1* | 1 | 3 | 20 | 0 | 2 | 4 | 0.230769 | 0.230769231 | 0.153846 | 45 |
| *eds1* | 2 | 4 | 18 | 1 | 1 | 2 | 0.181818 | 0.136363636 | 0.090909 | 45 |
| *eds1* | 3 | 5 | 14 | 1 | 0 | 0 | 0.066667 | 0 | 0 | 45 |
| *eds1* | 4 | 2 | 10 | 0 | 0 | 1 | 0.090909 | 0.090909091 | 0.090909 | 45 |
| *eds1* | 1 | 2 | 28 | 0 | 0 | 0 | 0 | 0 | 0 | 35 |
| *eds1* | 4 | 2 | 29 | 0 | 1 | 2 | 0.09375 | 0.09375 | 0.0625 | 35 |
| *eds1* | 5 | 3 | 17 | 0 | 1 | 1 | 0.105263 | 0.105263158 | 0.052632 | 35 |
| *eds1* | 7 | 5 | 33 | 2 | 1 | 1 | 0.108108 | 0.054054054 | 0.027027 | 35 |
| *eds1* | 8 | 4 | 20 | 0 | 2 | 2 | 0.166667 | 0.166666667 | 0.083333 | 35 |
| *eds1* | 1 | 4 | 9 | 0 | 1 | 4 | 0.357143 | 0.357142857 | 0.285714 | 38 |
| *eds1* | 2 | 4 | 11 | 0 | 1 | 3 | 0.266667 | 0.266666667 | 0.2 | 38 |
| *eds1* | 3 | 1 | 8 | 0 | 1 | 3 | 0.333333 | 0.333333333 | 0.25 | 38 |
| *eds1* | 4 | 2 | 8 | 0 | 0 | 2 | 0.2 | 0.2 | 0.2 | 38 |
| *eds1* | 6 | 3 | 11 | 0 | 0 | 3 | 0.214286 | 0.214285714 | 0.214286 | 38 |
| *eds1* | 7 | 4 | 19 | 0 | 0 | 2 | 0.095238 | 0.095238095 | 0.095238 | 38 |
| *ws* | 1 | 5 | 15 | 1 | 0 | 1 | 0.117647 | 0.058823529 | 0.058824 | 35 |
| *ws* | 2 | 5 | 27 | 0 | 0 | 1 | 0.035714 | 0.035714286 | 0.035714 | 35 |
| *ws* | 3 | 2 | 30 | 0 | 0 | 3 | 0.090909 | 0.090909091 | 0.090909 | 35 |
| *ws* | 4 | 4 | 23 | 0 | 2 | 3 | 0.178571 | 0.178571429 | 0.107143 | 35 |
| *ws* | 5 | 5 | 25 | 0 | 1 | 0 | 0.038462 | 0.038461538 | 0 | 35 |
| *ws* | 6 | 5 | 25 | 1 | 1 | 3 | 0.166667 | 0.133333333 | 0.1 | 35 |
| *ws* | 7 | 3 | 25 | 0 | 1 | 2 | 0.107143 | 0.107142857 | 0.071429 | 35 |
| *ws* | 8 | 3 | 26 | 0 | 0 | 2 | 0.071429 | 0.071428571 | 0.071429 | 35 |
| *ws* | 1 | 5 | 15 | 0 | 0 | 3 | 0.166667 | 0.166666667 | 0.166667 | 38 |
| *ws* | 2 | 5 | 14 | 0 | 1 | 3 | 0.222222 | 0.222222222 | 0.166667 | 38 |
| *ws* | 3 | 5 | 18 | 0 | 1 | 0 | 0.052632 | 0.052631579 | 0 | 38 |
| *ws* | 4 | 5 | 9 | 0 | 0 | 2 | 0.181818 | 0.181818182 | 0.181818 | 38 |
| *ws* | 5 | 3 | 4 | 0 | 1 | 0 | 0.2 | 0.2 | 0 | 38 |
| *ws* | 6 | 1 | 8 | 1 | 1 | 2 | 0.333333 | 0.25 | 0.166667 | 38 |
| *ws* | 4 | 5 | 33 | 2 | 3 | 4 | 0.214286 | 0.166666667 | 0.095238 | 39 |
| *ws* | 5 | 5 | 40 | 0 | 0 | 2 | 0.047619 | 0.047619048 | 0.047619 | 39 |
| *ws* | 6 | 4 | 19 | 0 | 3 | 3 | 0.24 | 0.24 | 0.12 | 39 |
| *ws* | 7 | 4 | 13 | 1 | 2 | 5 | 0.380952 | 0.333333333 | 0.238095 | 39 |
| *ws* | 8 | 2 | 24 | 0 | 0 | 7 | 0.225806 | 0.225806452 | 0.225806 | 39 |
| *ws* | 2 | 2 | 11 | 1 | 1 | 3 | 0.3125 | 0.25 | 0.1875 | 45 |
| *ws* | 3 | 1 | 13 | 0 | 1 | 2 | 0.1875 | 0.1875 | 0.125 | 45 |
| *ws* | 4 | 3 | 16 | 0 | 5 | 4 | 0.36 | 0.36 | 0.16 | 45 |
| ***Plant*** | **Bag** | **Position** | **Unattached** | **S3** | **S4.1** | **S4.2** | **S3 rate** | **S4.1 rate** | **S4.2 rate** | **Experiment** |
| *dcl1-7* | 1 | 3 | 22 | 0 | 0 | 0 | 0 | 0 | 0 | 24 |
| *dcl1-7* | 1 | 5 | 26 | 0 | 0 | 1 | 0.037037 | 0.037037037 | 0.037037 | 24 |
| *dcl1-7* | 2 | 4 | 35 | 0 | 2 | 3 | 0.125 | 0.125 | 0.075 | 24 |
| *dcl1-7* | 3 | 3 | 21 | 1 | 1 | 2 | 0.16 | 0.12 | 0.08 | 24 |
| *dcl1-7* | 5 | 1 | 18 | 0 | 2 | 0 | 0.1 | 0.1 | 0 | 24 |
| *dcl1-7* | 6 | 1 | 20 | 0 | 0 | 0 | 0 | 0 | 0 | 24 |
| *dcl1-7* | 6 | 4 | 7 | 1 | 1 | 0 | 0.222222 | 0.111111111 | 0 | 24 |
| *dcl1-7* | 1 | 3 | 22 | 0 | 1 | 0 | 0.043478 | 0.043478261 | 0 | 35 |
| *dcl1-7* | 3 | 3 | 23 | 1 | 0 | 4 | 0.178571 | 0.142857143 | 0.142857 | 35 |
| *dcl1-7* | 4 | 5 | 23 | 0 | 1 | 1 | 0.08 | 0.08 | 0.04 | 35 |
| *dcl1-7* | 5 | 2 | 22 | 0 | 0 | 2 | 0.083333 | 0.083333333 | 0.083333 | 35 |
| *dcl1-7* | 6 | 2 | 21 | 0 | 1 | 2 | 0.125 | 0.125 | 0.083333 | 35 |
| *dcl1-7* | 7 | 2 | 30 | 0 | 1 | 1 | 0.0625 | 0.0625 | 0.03125 | 35 |
| *dcl1-7* | 8 | 5 | 16 | 0 | 0 | 2 | 0.111111 | 0.111111111 | 0.111111 | 35 |
| *gai1* | 1 | 2 | 19 | 0 | 1 | 3 | 0.173913 | 0.173913043 | 0.130435 | 24 |
| *gai1* | 2 | 2 | 25 | 1 | 3 | 2 | 0.193548 | 0.161290323 | 0.064516 | 24 |
| *gai1* | 2 | 5 | 29 | 0 | 0 | 3 | 0.09375 | 0.09375 | 0.09375 | 24 |
| *gai1* | 3 | 4 | 15 | 0 | 0 | 0 | 0 | 0 | 0 | 24 |
| *gai1* | 4 | 1 | 5 | 0 | 0 | 0 | 0 | 0 | 0 | 24 |
| *gai1* | 5 | 5 | 26 | 1 | 1 | 0 | 0.071429 | 0.035714286 | 0 | 24 |
| *gai1* | 6 | 2 | 5 | 0 | 1 | 0 | 0.166667 | 0.166666667 | 0 | 24 |
| *gai1* | 3 | 4 | 11 | 0 | 0 | 2 | 0.153846 | 0.153846154 | 0.153846 | 35 |
| *gai1* | 4 | 3 | 20 | 1 | 4 | 3 | 0.285714 | 0.25 | 0.107143 | 35 |
| *gai1* | 1 | 3 | 11 | 0 | 0 | 2 | 0.153846 | 0.153846154 | 0.153846 | 38 |
| *gai1* | 2 | 3 | 15 | 0 | 1 | 1 | 0.117647 | 0.117647059 | 0.058824 | 38 |
| *gai1* | 3 | 4 | 14 | 0 | 0 | 2 | 0.125 | 0.125 | 0.125 | 38 |
| *gai1* | 4 | 1 | 14 | 0 | 1 | 1 | 0.125 | 0.125 | 0.0625 | 38 |
| *gai1* | 5 | 4 | 14 | 0 | 0 | 2 | 0.125 | 0.125 | 0.125 | 38 |
| *gai1* | 6 | 5 | 13 | 0 | 1 | 2 | 0.1875 | 0.1875 | 0.125 | 38 |
| *gai1* | 7 | 1 | 9 | 1 | 0 | 0 | 0.1 | 0 | 0 | 38 |
| *Ler* | 1 | 4 | 18 | 1 | 0 | 0 | 0.052632 | 0 | 0 | 24 |
| *Ler* | 2 | 1 | 25 | 0 | 1 | 1 | 0.074074 | 0.074074074 | 0.037037 | 24 |
| *Ler* | 2 | 3 | 39 | 0 | 3 | 2 | 0.113636 | 0.113636364 | 0.045455 | 24 |
| *Ler* | 3 | 2 | 19 | 0 | 0 | 0 | 0 | 0 | 0 | 24 |
| *Ler* | 4 | 3 | 18 | 0 | 2 | 2 | 0.181818 | 0.181818182 | 0.090909 | 24 |
| *Ler* | 4 | 5 | 8 | 1 | 1 | 0 | 0.2 | 0.1 | 0 | 24 |
| *Ler* | 5 | 2 | 15 | 0 | 3 | 2 | 0.25 | 0.25 | 0.1 | 24 |
| *Ler* | 5 | 4 | 26 | 0 | 0 | 0 | 0 | 0 | 0 | 24 |
| *Ler* | 6 | 5 | 25 | 0 | 1 | 2 | 0.107143 | 0.107142857 | 0.071429 | 24 |
| *ler* | 1 | 4 | 13 | 0 | 1 | 0 | 0.071429 | 0.071428571 | 0 | 35 |
| *ler* | 2 | 3 | 30 | 1 | 1 | 0 | 0.0625 | 0.03125 | 0 | 35 |
| *ler* | 3 | 1 | 19 | 0 | 0 | 1 | 0.05 | 0.05 | 0.05 | 35 |
| *ler* | 4 | 1 | 32 | 1 | 0 | 1 | 0.058824 | 0.029411765 | 0.029412 | 35 |
| *ler* | 5 | 4 | 25 | 0 | 1 | 1 | 0.074074 | 0.074074074 | 0.037037 | 35 |
| *ler* | 6 | 3 | 26 | 0 | 1 | 2 | 0.103448 | 0.103448276 | 0.068966 | 35 |
| *ler* | 7 | 4 | 20 | 0 | 0 | 0 | 0 | 0 | 0 | 35 |
| *ler* | 8 | 2 | 25 | 2 | 1 | 0 | 0.107143 | 0.035714286 | 0 | 35 |
| *Ler* | 1 | 2 | 22 | 0 | 0 | 1 | 0.043478 | 0.043478261 | 0.043478 | 38 |
| *Ler* | 3 | 3 | 5 | 1 | 2 | 3 | 0.545455 | 0.454545455 | 0.272727 | 38 |
| *Ler* | 4 | 3 | 12 | 0 | 2 | 2 | 0.25 | 0.25 | 0.125 | 38 |
| *Ler* | 6 | 4 | 12 | 1 | 3 | 3 | 0.368421 | 0.315789474 | 0.157895 | 38 |
| *Ler* | 7 | 5 | 7 | 0 | 1 | 0 | 0.125 | 0.125 | 0 | 38 |
