# Supplementary material for: Multiple immunity-related genes control susceptibility of Arabidopsis thaliana to the parasitic weed Phelipanche aegyptiaca
Source: PeerJ. 2020 Jun 8;8:e9268. doi: 10.7717/peerj.9268 (PMC7289146; doi:10.7717/peerj.9268)
Supplement: Supplemental Information 4 [file peerj-08-9268-s004.docx]

**Supplementary Table 4.** Total number of mutant lines more or less susceptible than wildtype *A. thaliana* to *P. aegyptiaca* parasitization.

|  |  | **95% confidence level** | | |  | **99% confidence level** | | |
| --- | --- | --- | --- | --- | --- | --- | --- | --- |
|  | **More susceptible** | | **No difference** | **Less susceptible** | **More susceptible** | | **No difference** | **Less susceptible** |
| **Initial Attach.** | 2 | | 43 | 2 | 2 | | 45 | 0 |
| **Early tubercle** | 2 | | 31 | 14 | 1 | | 38 | 8 |
| **Late tubercle** | 2 | | 29 | 16 | 0 | | 38 | 9 |
